# The seasonal sea-ice zone in the glacial Southern Ocean as a carbon sink

**DOI:** 10.1038/ncomms9136

**Published:** 2015-09-18

**Authors:** Andrea Abelmann, Rainer Gersonde, Gregor Knorr, Xu Zhang, Bernhard Chapligin, Edith Maier, Oliver Esper, Hans Friedrichsen, Gerrit Lohmann, Hanno Meyer, Ralf Tiedemann

**Affiliations:** 1 Alfred Wegener Institute Helmholtz Centre for Polar and Marine Research, Am Alten Hafen 26, Bremerhaven 27568, Germany; 2 Cardiff School of Earth and Ocean Sciences, Cardiff, Wales CF10 3AT, UK; 3 Free University of Berlin, Berlin 14195, Germany

## Abstract

Reduced surface–deep ocean exchange and enhanced nutrient consumption by phytoplankton in the Southern Ocean have been linked to lower glacial atmospheric CO_2_. However, identification of the biological and physical conditions involved and the related processes remains incomplete. Here we specify Southern Ocean surface–subsurface contrasts using a new tool, the combined oxygen and silicon isotope measurement of diatom and radiolarian opal, in combination with numerical simulations. Our data do not indicate a permanent glacial halocline related to melt water from icebergs. Corroborated by numerical simulations, we find that glacial surface stratification was variable and linked to seasonal sea-ice changes. During glacial spring–summer, the mixed layer was relatively shallow, while deeper mixing occurred during fall–winter, allowing for surface-ocean refueling with nutrients from the deep reservoir, which was potentially richer in nutrients than today. This generated specific carbon and opal export regimes turning the glacial seasonal sea-ice zone into a carbon sink.

## Abstract (web summary)

Lower glacial atmospheric CO_2_ has been linked to enhanced carbon storage in the Southern Ocean, yet the associated biological and physical mechanisms are poorly understood. Here, the authors combine diatom and radiolarian isotope measurements, and model simulations to investigate surface–subsurface processes.

## Graphical abstract

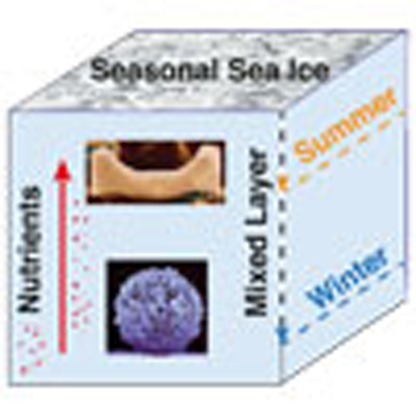

The past four climate cycles are characterized by a repetitive pattern of gradually declining and rapidly increasing atmospheric CO_2_ concentrations, ranging between ∼180 p.p.m. during glacials and ∼280 p.p.m. during interglacials^1^. Although multiple processes on land and in the ocean are involved in the modulation of the observed CO_2_ variability^2^, physical and biological processes in the Southern Ocean (SO) have been identified to be the key in these changes^3^. This view is supported by the tight relationship between CO_2_ and Antarctic temperature development^4^. Most important are changes in ocean ventilation/stratification, sea-ice extent, wind patterns, atmospheric transport of micronutrients (for example, iron) and biological productivity and export, according to proxy and model-based studies^3^,^5^,^6^,^7^,^8^,^9^,^10^,^11^. Despite the scientific progress, the different hypotheses on the SO's sensitivity to modulate the carbon cycle and the identification of involved processes remain under debate. In the SO, the availability of silicon nutrients (Si), the consumption by primary producers (diatoms) and cycling pathways are key for effective carbon sequestration^8^,^12^. The widespread deposition of biogenic opal, which consists primarily in diatoms but also in radiolarians and, to a minor extent, in sponge spicules, allows for the application of specific opal-based proxies to trace these processes and related environmental conditions. However, controversial views exist for the interpretation of the proxies used to trace past productivity and their impact on the carbon cycle^3^,^5^,^13^. Similarly, glacial–interglacial changes in surface ocean stratification, which control ocean atmosphere exchange and the availability of nutrients, have been discussed contentiously. This has resulted in different notions of the impact of physical and biological processes in ice-free and ice-covered areas on the glacial–interglacial climate evolution^3^,^5^,^13^. Isotope records of diatom-bound nitrogen (δ^15^N) are interpreted to indicate a low-productivity glacial seasonal sea-ice zone (SIZ) resulting from constricted nutrient supply to the surface ocean, owing to permanent and enhanced near-surface stratification^3^,^13^. Further information on surface water (euphotic zone) conditions comes from oxygen isotopes (δ^18^O) of diatoms, used to identify meltwater supply from the Antarctic continent^14^,^15^,^16^,^17^. Silicon isotope (δ^30^Si) measurements on diatoms and sponge spicules provide insights into the development of silicon utilization in surface waters^18^,^19^,^20^ and the silicon inventory of the deep ocean^19^,^21^,^22^. A yet unexploited window into subsurface and deeper water conditions presents the isotope signal from radiolarians (protozooplankton). In combination with the diatom isotope data, these signals provide an enhanced framework to detect changes of upper and lower water column conditions, and thus the pattern and glacial–interglacial variability of stratification and nutrient exchange.

Here we apply the new approach to combine δ^18^O and δ^30^Si measurements of diatom and radiolarian opal to two late Quaternary sediment cores (PS1768-8 and PS1778-5) from the sea ice-free Antarctic Zone and from the Polar Front Zone of the Atlantic sector of the SO, respectively (Fig. 1). We are aware that the calibration of the new proxies requires further investigations, especially with respect to the isotope fractionation of radiolarians. Here we attenuate the lack of data on radiolarian fractionation by combining δ^30^Si measurements from surface sediments and water-column samples available from the study area. As least information is available on oxygen isotope fractionation of radiolarians, we primarily base our δ^18^O-related interpretations on the signals from diatom opal. Altogether, the combination of our opal isotope results with other proxy data and climate simulations using a fully coupled global atmosphere–ocean general circulation model^23^ (AOGCM) enables the establishment of coherent paleoceanographic scenarios. This combined data/modelling interpretation implies that the glacial near-surface stratification in the SIZ was variable. Relatively deep mixing during fall and winter allowed for surface-ocean refueling with nutrients from a potentially enriched deep reservoir, which generated a carbon sink in the glacial SIZ.

## Results

### Opal-based isotope proxies

A critical requirement for appropriate analyses and interpretation of δ^18^O and δ^30^Si in diatom (δ^18^O_diat_ and δ^30^Si_diat_) and radiolarian (δ^18^O_rad_ and δ^30^Si_rad_) opal is the extraction and separation of both microfossil groups (Methods, Supplementary Figs 1–4 and Supplementary Table 1). Our diatom fraction (10–40 μm) used for isotope measurements to reconstruct surface water conditions is dominated by two species: *Eucampia antarctica* in the lower part of the cores and *Thalassiosira lentiginosa* in the upper core portions. The shift in species composition is abrupt and its timing is unrelated to the glacial–interglacial change of the opal isotope signals (Methods and Supplementary Fig. 5), which suggests that the diatom isotope signals are not biased by species-related effects. Except for two studies from the North Pacific, this is in line with other investigations, indicating vital effects to be either non-existent or within the analytical reproducibility^14^,^17^,^24^. In contrast to the diatoms, the species composition of the individual radiolarian fractions does not significantly vary throughout the investigated core sections, so that species-related isotope effects in the different fractions remain unlikely. The radiolarian fraction >250 μm (PS1768-8) consists of two large-sized species (*Actinomma antarctica* and *Spongotrochus glacialis* adult), mainly dwelling in the upper 100–400 m of the water column, thus representing surface–subsurface conditions (Supplementary Table 2). Radiolarians assembled in the 125- to 250- μm fraction (PS1768-8) and the >125-μm fraction (PS1778-5) display a more diverse species composition, also including species with a deeper habitat (>400 m)^25^ (Methods). We also rule out seasonal effects, because sediment trap studies show that the diatom and radiolarian export in the SIZ of the SO occur synchronously and are restricted to spring–summer^26^.

**Figure 1:**
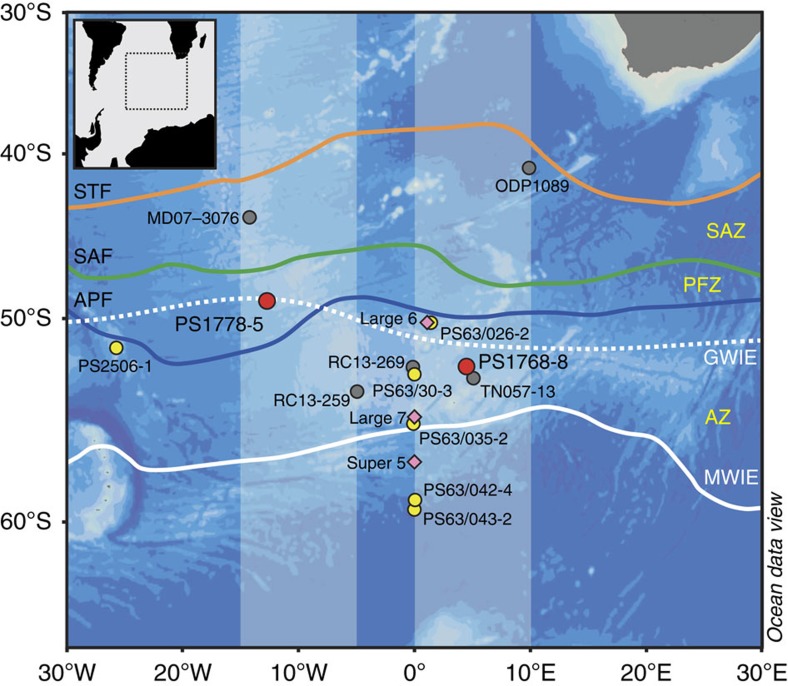
Map of the study area. The map shows the location of sediment core PS1768-8 in the sea-ice free Antarctic Zone (AZ) of the Atlantic sector of the Southern Ocean and core PS1778-5 from the Polar Front Zone (PFZ). Also indicated are sites of water column sampling^29^ (pink diamonds), surface sediment sampling (yellow dots), and locations of cores discussed in the text (grey dots). Locations of the modern winter ice edge (MWIE) and the GWIE were derived from data in refs 34, 67, respectively. Oceanic fronts from ref. 68: Antarctic Polar Front (APF), Subantarctic Front (SAF) and Subtropical Front (STF); the latter two delimit the Subantarctic Zone (SAZ). Light-blue-shaded areas represent the zones of modelled transects (Fig. 5b–e).

**Figure 2:**
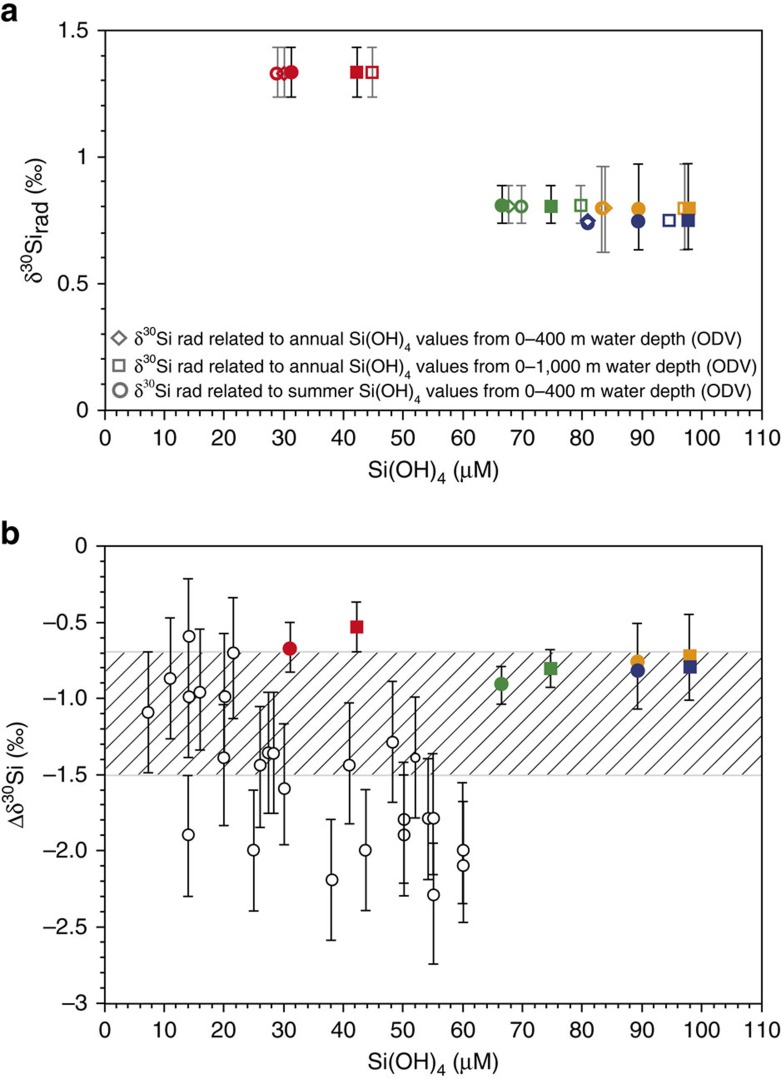
δ^30^Si data of radiolarians (δ^30^Si_rad_) and Δδ^30^Si offsets in surface sediments compared with Si(OH)_4_ concentrations and to Δδ^30^Si offsets of diatoms. (**a**) δ^30^Si data of radiolarians (δ^30^Si_rad_) in four surface sediments from the study area compared with Si(OH)_4_ concentrations in sea water. Colours refer to different surface sediments (red: PS63/026-2, green: PS63/035-2, blue: PS63/042-2, orange: PS63/043-2) (for site location, see Fig. 1 and Supplementary Table 3). Filled circles and squares present the δ^30^Si_rad_ values (Supplementary Table 3) plotted versus Si(OH)_4_ concentrations measured at different water depth intervals (filled circles: upper ∼300–400 m and filled squares: upper 1,000 m) at nearby seawater sampling stations^29^ (Fig. 1 and Supplementary Table 3), whereas the open symbols display the δ^30^Si_rad_ values plotted versus annual and summer Si(OH)_4_ concentrations at different water depth intervals in the study area according to the World Ocean Atlas^33^ (Supplementary Table 3). (**b**) Δδ^30^Si offsets between δ^30^Si values of seawater samples^27^,^28^,^29^,^69^ and δ^30^Si values of radiolarians (δ^30^Si_rad_) and diatoms (δ^30^Si_diat_), respectively, versus seawater Si(OH)_4_ concentrations at the seawater sampling stations. Coloured symbols display different surface sediments (see Fig. 2a). Different symbols (circles and squares) display the Δδ^30^Si offsets between δ^30^Si_rad_ values of the four surface sediment samples and seawater δ^30^Si values at different water depth intervals (filled circles: upper ∼300–400 m and filled squares: upper 1,000 m, see Fig. 2a) at the nearby oceanographic stations^29^ The dashed area illustrates the diatom δ^30^Si fractionation offset of −1.1±0.4‰ obtained from diatom culture studies^24^. The white dots with error bars display the Δδ^30^Si offsets between δ^30^Si_diat_ and δ^30^Si of seawater obtained from field data^27^,^28^,^29^,^69^. Error bars show ±2*σ* s.d.

The δ^18^O signal in diatoms generally depends on both temperature and δ^18^O in seawater^17^. A robust relationship between diatom δ^30^Si and silicic acid utilization is derived from culture experiments and field data^19^,^20^,^24^,^27^,^28^,^29^,^30^. Diatom culture studies point to a mean fractionation factor of −1.1‰ (ref. 24) and show limited variation with species and growth rate^19^. Larger variability in diatom δ^30^Si fractionation was derived from field data ranging between approximately −0.6‰ and −2.3‰, which reflects the natural variation but also the methodological challenge of calculating fractionation offsets^19^ (Fig. 2b). Although the oxygen and silicon isotope fractionation of diatoms is rather well investigated, the knowledge concerning the isotope fractionation of radiolarians is less developed. The generation of such data is complicated by the lack of successful radiolarian culturing experiments^31^ and isotope measurements of radiolarians collected in the water column. A first approach to obtain information on this important issue relies on the modelling of a fractionation offset ranging between −1.1‰ and −2.1‰ derived from deglacial δ^30^Si_rad_ values^32^. To assist the interpretation of our results, we moved a step forward and estimated the radiolarian fractionation offset (Δδ^30^Si_rad_) by using δ^30^Si_rad_ values from four surface sediment samples from the Atlantic sector in combination with δ^30^Si_Si(OH)4_ values from surface and deeper waters close to the surface sediment sample sites^29^ (Figs 1, 2a,b, and Supplementary Table 3). Considering that the fractionation of diatoms and sponges is suggested to occur in equilibrium with the surrounding water^19^,^24^, it is reasonable to assume that this is also true for the fractionation of radiolarians. For the calculation of Δδ^30^Si_rad_, we used the following equation adapted from ref. 21


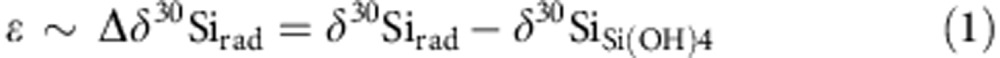


where *ɛ* is the fractionation factor by opal-producing organisms, δ^30^Si_rad_ is the silicon isotope composition of radiolarian opal and δ^30^Si_Si(OH)4_ is the silicon isotope composition of sea water. The Δδ^30^Si_rad_ values were calculated with δ^30^Si_Si(OH)4_ values averaged from two different water depth intervals (0 to ∼300–400 m and 0 to ∼1,000 m), to cover all possible depth ranges of the included species (Fig. 2b and Supplementary Table 3). The obtained Δδ^30^Si_rad_ values range between −0.5‰ and −0.9‰, and show a linear relationship with the Si(OH)_4_ concentrations. The δ^30^Si_rad_ fractionation offset calculated in our study is more positive than the fractionation applied in ref. 32 (−1.1‰ to −2.1‰). However, both fractionation estimates are in the range of the observed diatom fractionation (Fig. 2b). The modern δ^30^Si_rad_ values display an inverse trend to the Si(OH)_4_ concentrations^29^,^33^ in the upper 400 and upper 1,000 m of the water column (Fig. 2a and Supplementary Table 3). Higher δ^30^Si_rad_ values of approximately +1.4‰ correspond to lower Si(OH)_4_ concentrations of 30–45 μM l^−1^, whereas lower δ^30^Si_rad_ values of +0.7‰ and +0.8‰ correlate with higher Si(OH)_4_ concentrations of 67–98 μM l^−1^, which is comparable to the relationship between δ^30^Si values and Si(OH)_4_ concentrations documented for diatoms and sponges.

**Figure 3:**
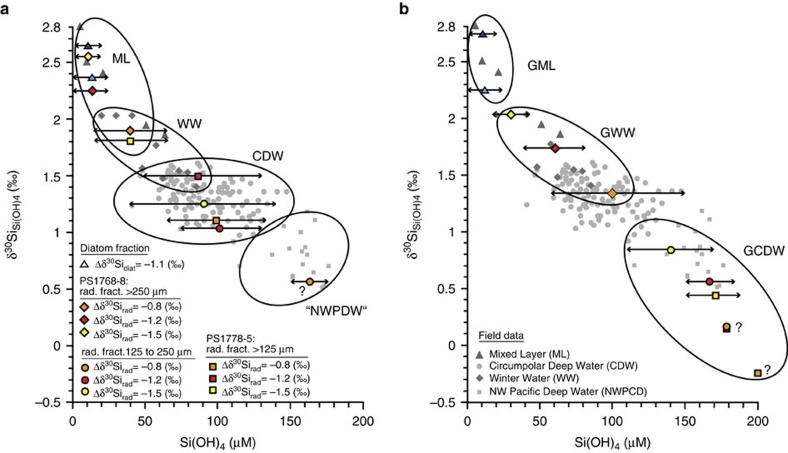
Reconstructed δ^30^Si_Si(OH4)_ values and Si(OH)_4_ seawater concentrations compared with values in modern water masses. (**a**) Average Holocene and (**b**) glacial-time δ^30^Si_Si(OH4)_ values and Si(OH)_4_ concentrations in seawater reconstructed from δ^30^Si_rad_ data and δ^30^Si_diat_ values in cores PS1768-8 and PS1778-5 compared with modern seawater δ^30^Si_Si(OH4)_ values and Si(OH)_4_ concentrations in different water masses^29^ (Supplementary Table 4). The blue triangles indicate the δ^30^Si_Si(OH4)_ values reconstructed from the δ^30^Si_diat_ data of the two cores by using a Δδ^30^Si offset of −1.1‰ (light blue: core PS1768-8 and dark blue: core PS1778-5), the coloured diamonds indicate the δ^30^Si_Si(OH4)_ values reconstructed from the δ^30^Si_rad_ data of the size fraction >250 μm in core PS1768-8, the coloured dots indicate the δ^30^Si_Si(OH4)_ values reconstructed from the δ^30^Si_rad_ data of the size fraction 125–250 μm in core PS1768-8 and the coloured squares indicate the δ^30^Si_Si(OH4)_ values reconstructed from the δ^30^Si_rad_ data of the size fraction >125 μm in core PS1778-5. The Δδ^30^Si offsets applied to the δ^30^Si_rad_ data were −0.8‰ (orange), −1.2‰ (red) and −1.5‰ (yellow) (see Methods). The obtained δ^30^Si_Si(OH)4_ values were related to δ^30^Si_Si(OH)4_ values from water stations and to their ranges in Si(OH)_4_ concentrations^20^,^28^,^29^. The modern δ^30^Si values and Si(OH)_4_ concentrations were measured on seawater samples from the mixed layer (ML; grey triangles)^29^, Winter Water (WW; grey diamonds)^29^, CDW (grey dots)^28^,^29^ and Northwest Pacific Deep Water (‘NWPDW'; grey squares)^20^. The reconstructed glacial-time δ^30^Si_Si(OH4)_ values and Si(OH)_4_ concentrations (**b**) may allow to discriminate Glacial Mixed Layer (GML), Glacial WW (GWW) and Glacial CDW (GCDW). Double-headed arrows display the range in Si(OH)_4_ concentrations that can be attributed to the reconstructed δ^30^Si_Si(OH4)_ values. It is worth noting that δ^30^Si_Si(OH4)_ estimations based on Δδ^30^Si_rad_ of −0.8‰ may tend to overestimated Si(OH)_4_ concentrations.

To test the reliability of the available information on diatom and radiolarian fractionation we calculated δ^30^Si_Si(OH)4_ from δ^30^Si values of radiolarians and diatoms averaged over the Holocene in both cores (Methods and Supplementary Table 4) and related the obtained δ^30^Si_Si(OH)4_ data to δ^30^Si_Si(OH)4_ and Si(OH)_4_ concentrations reported from modern water column studies^29^ (Fig. 3a). In our δ^30^Si_Si(OH)4_ calculation we considered a fractionation of −1.1‰ for diatom δ^30^Si data^24^. Considering the remaining uncertainty in the definition of radiolarian fractionation offsets, we tested the applicability of three offset values. This includes Δδ^30^Si_rad_ of −0.8‰ (average estimated offset from this study, Supplementary Tables 3 and 4), −1.5‰ (average estimated offset from ref. 32) and −1.2‰ representing an average over both. We note that the Holocene δ^30^Si_Si(OH)4_ values reconstructed from δ^30^Si of diatoms and surface–subsurface dwelling radiolarians (>250 μm fraction) are in the range of δ^30^Si_Si(OH)4_ values reported from the modern mixed layer (ML) in the Atlantic sector of the SO^29^ (Fig. 3a). The δ^30^Si_Si(OH)4_ values reconstructed from the δ^30^Si data in the radiolarian fractions 125–250 μm and >125 μm, which also include species with a deeper habitat, are shifted towards lower values. These δ^30^Si_Si(OH)4_ values are in the range of δ^30^Si_Si(OH)4_ data and Si(OH)_4_ concentrations from the Circumpolar Deep Water (CDW)^28^,^29^ (Fig. 3a). While the application of Δδ^30^Si_rad_ values of −1.2‰ and −1.5‰ leads to realistic seawater Si(OH)_4_ concentrations, estimates calculated with a fractionation offset of -0.8‰ tend to result in overestimated Si(OH)_4_ concentration. This is most apparent for the result from the 125- to 250-μm radiolarian fraction (PS1768-8) reaching values comparable to those in modern Northwest Pacific Deep Water^20^, which exceed CDW concentrations (Fig. 3a). Although information on isotope fractionation in radiolarians remains incomplete and requires additional efforts (for example, in water column studies and new approaches for radiolarian culturing), our Δδ^30^Si_rad_ calculations and their relation to modern Si(OH)_4_ concentrations point to a similar fractionation in diatoms and radiolarians. This assumption represents a step towards quantification of past Si(OH)_4_ concentration and its variability in surface and subsurface to intermediate-deeper water.

### Down-core data interpretation

**Figure 4:**
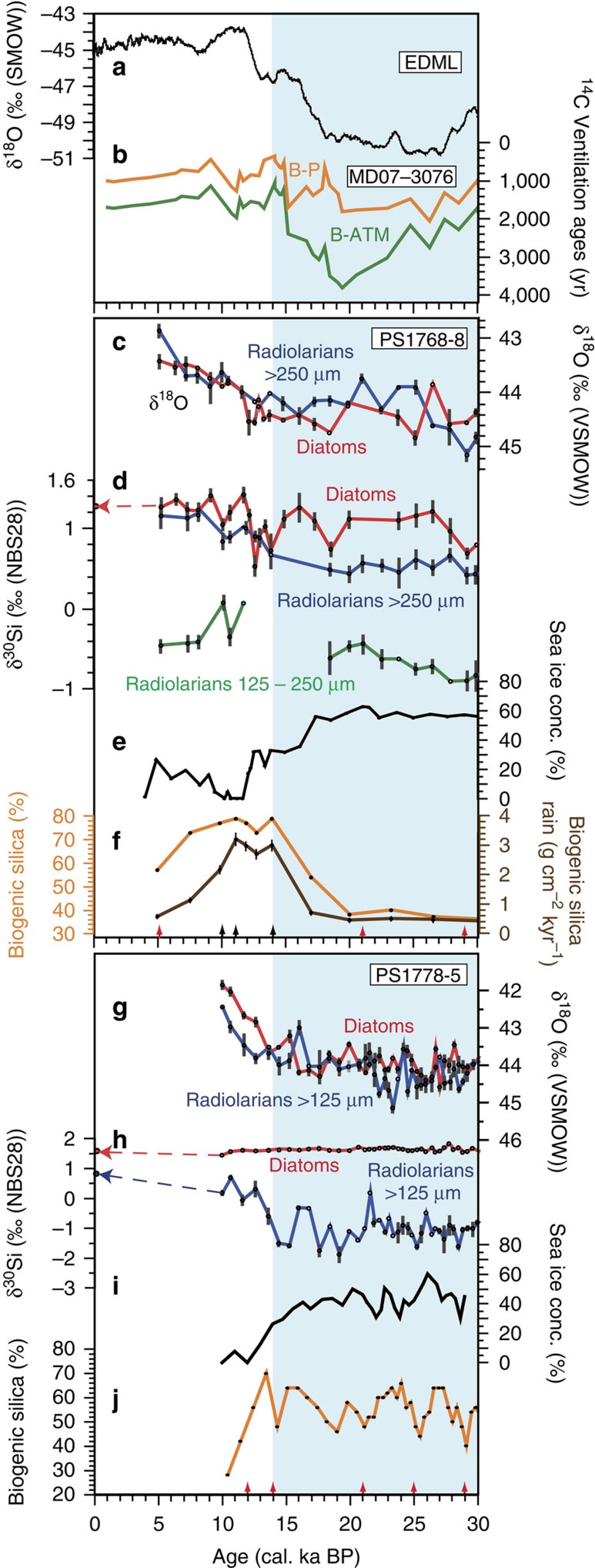
Oxygen and silicon isotope records of diatoms and radiolarians from cores PS1768-8 and PS1778-5 compared with other paleoclimatic records over the last 30 kyr. (**a**) δ^18^O record of Antarctic ice core drilled at EPICA Dronning Maud Land (EDML) site^70^ against AICC2012 (Antarctic Ice Core Chronology 2012)^62^. (**b**) ^14^C ventilation ages plotted as benthic-planktic foraminifera age difference (B-P) and benthic-atmospheric difference (B-ATM) from core MD07-3076 (Fig. 1)^57^. (**c**–**f**) Proxies from core PS1768-8 located in the glacial SIZ. (**c**) δ^18^O and (**d**) δ^30^Si records from one diatom and two radiolarian fractions (>250 μm and 125–250 μm) measured at the same aliquot of biogenic opal. The red dashed arrow points to the δ^30^Si_diat_ value obtained from a nearby seafloor surface-sediment sample assumed to be of modern age (Supplementary Table 7). Error bars indicate range of replicate and triplicate measurements (Supplementary Tables 8–12). (**e**) Diatom transfer function-based estimates of WSIC^35^. (**f**) Biogenic silica percentages and biogenic silica rain rates^50^. (**g**–**j**) Proxies from core PS1778-5 located close to the GWIE. (**g**) δ^18^O and (**h**) δ^30^Si records of diatoms and radiolarians (>125 μm fraction) measured at the same aliquot of biogenic opal. The red and blue dashed arrows point to the δ^30^Si_diat_ and δ^30^Si_rad_ values obtained from a nearby seafloor surface sediment sample assumed to be of modern age (Supplementary Table 7) (**i**) Diatom transfer function-based estimates of WSIC. (**j**) Biogenic silica percentages. Arrows in **f** and **j** indicate age pointers (black arrows mark AMS^14^C dates and red arrows mark ages obtained by diatom and radiolarian biofluctuation stratigraphy, see Supplementary Tables 5 and 6). Blue-shaded area delineates Marine Isotope Stage (MIS) 2 and the late part of MIS 3.

During the last glacial, core PS1768-8 (52°35.61′S, 4°28.5′E, water depth 3,270 m) was positioned in the northern glacial SIZ and core site PS1778-5 (49°00.7′S, 12°41.8′W, water depth 3,380 m) was in the area of the glacial winter sea-ice edge (GWIE)^34^ (Fig. 1 and Supplementary Fig. 6). This is in agreement with glacial-time winter sea-ice concentrations (WSICs) based on a new transfer function^35^, which display glacial sea-ice concentrations ∼60% in the PS1768-8 record (Fig. 4e) and ∼40% at PS1778-5 (Fig. 4i). We assigned sea-ice concentrations of 40%–50% to be indicative of the average paleo-sea-ice edge, because these values are in the middle of the abrupt decline of Antarctic sea-ice concentration, which marks the modern sea-ice edge^35^,^36^. A similar definition of the average sea-ice edge was proposed based on microwave remote-sensing observations^37^. Our sediments document the last glacial, the glacial–interglacial transition and the early part of the Holocene (Fig. 4). In the absence of biogenic carbonate, which hampers the development of continuous foraminiferal oxygen isotope records and carbonate-based AMS^14^C data series in the studied cores, the generation of age models for both cores considers the dating strategy and stratigraphic data from a compilation of last glacial sea-surface temperature and sea-ice records from the Atlantic sector of the SO^38^ (Methods and Supplementary Figs 7,8).

In both cores, PS1768-8 and PS1778-5, the δ^18^O_diat_ and δ^18^O_rad_ signals display a similar pattern with decreasing values from the last glacial period to the early Holocene (Fig. 4c,g). The δ^18^O_diat_ and δ^18^O_rad_ values range between +45.1‰ and +41.7‰, thus being close to the δ^18^O_diat_ values obtained from other SO sediment cores^14^,^15^,^16^. The major shifts from glacial to Holocene δ^18^O values occur in close relation to sea-surface water temperature (SST) increase overprinting the ice volume signal (Supplementary Figs 7 and 8). The early–middle Holocene diatom and radiolarian δ^18^O records from PS1768-8 display a trend similar to a planktic foraminifer δ^18^O record from the nearby core TN057-13 (ref. 15, Fig. 1 and Supplementary Fig. 7). At both sites, our δ^18^O_diat_ records are inconsistent with distinct glacial freshwater supply resulting from iceberg melting. This differs from the δ^18^O_diat_ records reported from cores TN057-13 (ref. 15) and RC13-259 (ref. 14) recovered in our study area (Fig. 1). These records are characterized by generally lower glacial values and higher δ^18^O_diat_ values during the last deglaciation and the Holocene. In TN057-13, the δ^18^O_diat_ record is punctuated by δ^18^O_diat_ decreases that are in close correlation with increased values of ice rafted debris (IRD)^15^. According to a more recent geochemical study, IRD in TN057-13 primarily consists of volcanic tephra from the South Sandwich Islands transported by sea ice to the studied site^39^. Measurements of δ^18^O on samples that contain, besides biogenic silica, also non-biogenic components such as terrigenous minerals and volcanic tephra, may result in lower δ^18^O values and thus bias the isotopic signal towards freshwater-related values^17^,^40^. Therefore, the apparent anti-correlation between the content of IRD (mostly tephra) and δ^18^O_diat_ values in core TN057-13 may not only be explained by salinity decrease due to iceberg melting but also by a contribution from δ^18^O-depleted tephra to the δ^18^O_diat_ signal. In contrast, IRD deposition in core PS1768-8 decreases between 18 and 16 cal. ka BP. Thus, the δ^18^O_diat_ values show no correlation with IRD (Supplementary Fig.7), which suggests that the δ^18^O_diat_ data presented here may be more reliable than those of ref. 15. This is confirmed by the purity of the cleaned samples in this study, which is exceptionally high, ranging between 97.9% and 99.8% SiO_2_ (Methods and Supplementary Table 1), ruling out bias of our δ^18^O_diat_ values.

The δ^30^Si signal of diatoms in core PS1768-8 ranges generally around +1‰ in the glacial and increases to about +1.2‰ in the Holocene (Fig. 4d). The values correspond well to those reported from the nearby core RC13-269 (ref. 41 and Fig. 1). In the northern core PS1778-5, we observe distinctly higher glacial δ^30^Si_diat_ values around +1.6‰ and a slight decrease by ∼0.05‰ towards the Holocene, which is however within the analytical error (Fig. 4h).

In comparison with the diatom records, the contrasts between glacial and interglacial δ^30^Si_rad_ values are generally more pronounced. During glacial conditions, the silicon isotope signals of diatoms and radiolarians in core PS1768-8 display offsets, with ∼0.6‰ lower δ^30^Si_rad_ values in the >250 μm fraction that represents surface–subsurface conditions and ∼1.7‰ lower δ^30^Si_rad_ values in the 125- to 250-μm fraction, which also includes signals from intermediate-deeper waters. Even more pronounced is the offset between the glacial diatom and radiolarian δ^30^Si signals from the northern core PS1778-5 where the radiolarian fraction >125 μm combines species dwelling at surface–subsurface and intermediate-deeper water depth (Methods). The glacial δ^30^Si_rad_ values range around −1.1‰ and thus are ∼2.7‰ lower than the diatom values. During the deglacial transition, surface–subsurface δ^30^Si_rad_ values in the southern core PS1768-8 increase to δ^30^Si_diat_ values and remain close to the diatom values during the Holocene (Fig. 4d). Such deglacial convergence of the diatom and radiolarian records is also observed in core PS1778-5, but an offset of ∼0.9‰ between δ^30^Si_diat_ and δ^30^Si_rad_ persists into the early Holocene (Fig. 4h).

We interpret the glacial offsets between the δ^30^Si_diat_ and δ^30^Si_rad_ records as resulting from the presence of a glacial surface-water stratification separating the diatom and radiolarian habitats at the time of their production (spring–summer). Considering that the δ^18^O_diat_ records are not indicative of significant freshwater supply, we suggest that the stratification is primarily induced by sea-ice melt during spring, as melting sea ice has no significant effect on the oxygen isotopic composition of surface waters^42^ but largely affects surface ocean salinity and thus the surface water structure even beyond the winter sea-ice edge. The effect of sea-ice melting during spring is assisted by seasonal warming and weakening winds as observed in the modern SO. Assuming that the offsets between the δ^30^Si_diat_ and δ^30^Si_rad_ records reflect stratification in the upper water column, they would point to glacial surface waters with increased silicic acid consumption and subsurface and intermediate deepwaters with higher silicic acid availability. The deglacial convergence between the δ^30^Si_diat_ and δ^30^Si_rad_ records may point to a deepening of the spring–summer ML depth (MLD), leading to the same Si pool for surface–subsurface-dwelling radiolarians (>250 μm fraction) and surface-dwelling diatoms at site PS1768-8. Considering that this interpretation is strongly based on a radiolarian isotope signal obtained from a nearly monospecific >250 μm fraction, we are confident that the δ^30^Si_rad_ signal represents environmental change rather than fractionation effects.

### Modelling glacial sea ice and MLD variability

To further evaluate the physical changes associated with sea-ice variations, we re-analysed two model simulations with conditions during the Last Glacial Maximum (LGM)^23^ and interglacial periods^43^, respectively, and performed two additional sensitivity experiments to test the impact of a deglacial CO_2_ rise and a poleward wind field shift in the SO. All model results are based on simulations using the same fully coupled AOGCM^23^. The model configuration includes the atmosphere component ECHAM5 (ref. 44) at T31 resolution (∼3.75°) with 19 vertical layers, complemented by a land-surface scheme including dynamical vegetation (JSBACH)^45^. The ocean component MPI-OM^46^, including the dynamics of sea ice formulated using viscous-plastic rheology^47^, has an average horizontal resolution of 3° × 1.8° with 40 uneven vertical layers. The performance of this climate model was evaluated for SO Holocene^43^ and glacial^23^ conditions, showing that the glacial and interglacial (pre-industrial) sea-ice field (Supplementary Fig. 9) and general ocean circulation can be simulated reasonably well, providing a suitable reference to explore the underlying physical mechanism accounting for the proxy records developed in this study. The model has also been applied to analyse glacial millennial-scale variability^48^ and warm climates in the Miocene^49^. For further details of the model and experimental configuration, see Methods and Supplementary Figs 9–11.

**Figure 5:**
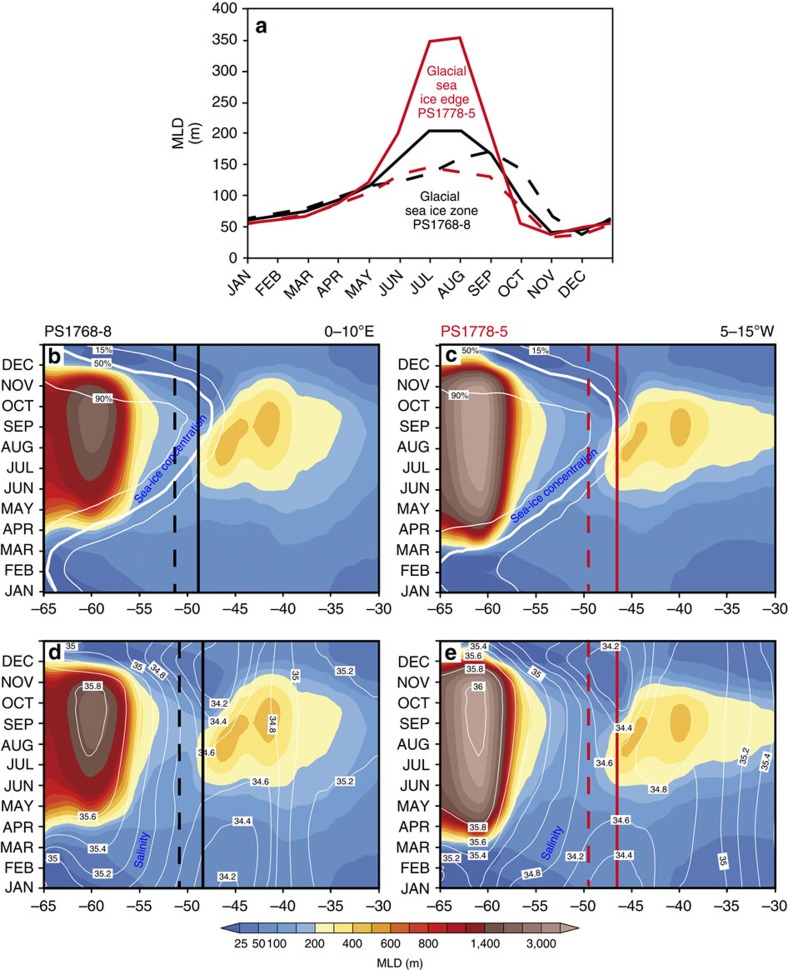
Modelled monthly averaged MLD, sea-ice concentration and salinity in the Atlantic sector of the Southern Ocean during the LGM. (**a**–**e**) Monthly averaged MLDs for PS1768-8 and PS1778-5 overlaid in **b** and **c** with sea-ice concentration (%), and in **d** and **e** with sea-surface salinity (psu). Shown are latitudinal transects zonally averaged between 0 and 10°E (for PS1768-8), and 5 and 15°W (for PS1778-5). Dashed lines indicate the respective changes at the exact latitudinal positions of the cores, whereas the solid lines show the changes at the latitudinal position, where the physical conditions coincide with the reconstructed proxy-based LGM WSIC. The transect zone and core locations are indicated in Fig. 1. The relatively deep glacial MLD at *ca*. 60–65°S in the Weddell Sea (**b**–**e**) are mainly attributed to strengthened brine rejection associated with enhanced *in-situ* sea-ice formation and northward sea-ice export, which are key processes for SO deep water formation during the LGM (for example, see ref. 23).

By re-analysing model results for the LGM^23^ from two latitudinal transects (centred at the respective core locations) across the SO in 10° longitudinal windows (Fig. 1), we identify strong seasonal changes in sea-ice cover in the area of both core locations (Fig. 5b,c) consistent with the interpretation of a link between vertical stratification changes and seasonal sea-ice variations. These variations result in annual sea-surface salinity and accompanying MLD variations that favour an increase of the MLD during the season of sea ice growth, followed by an MLD decrease when sea ice declines. Hence, the glacial simulation shows a relatively shallow glacial ML during austral spring–summer, reaching minimum values of 40–60 m in the study area (Fig. 5), which can be attributed to the melting of sea ice during this time. Such a pattern would separate the main habitat depth of diatoms from the deeper living radiolarians as suggested by the glacial diatom-radiolarian δ^30^Si offset (Fig. 4d,h). An MLD deepening is simulated for fall–winter seasons, which is promoted by enhanced vertical mixing during sea-ice formation (Fig. 5).

To test the robustness of this interpretation, zonal heterogeneities in sea-ice distribution need to be taken into account. Therefore, we evaluated the seasonal MLD changes for both the exact latitudes of the core locations (Fig. 5, dashed lines) and the latitudes where the physical conditions coincide with the proxy-based LGM WSIC (Fig. 5, solid lines). The respective WSIC amounts on average to 60% at PS1768-8 and to 41.6% at PS1778-5 (Supplementary Fig. 6). This approach ties the proxy-based information from the sediment cores to physical conditions simulated by the model. Based on this approach, we can estimate that during glacial spring–summer the MLD reached minimum values between 40 and 60 m at both studied sites and increased during sea-ice formation in fall–winter, reaching a maximum depth of up to 350 m at the site located in the area of the GWIE (Fig. 5a).

## Discussion

The combination of proxy data and AOGCM modelling implies that the sea surface of the glacial SIZ and the GWIE, at least in the Atlantic sector, was distinctly stratified during austral spring–summer with a relatively shallow MLD (40–60 m; Fig. 5). Increased glacial deposition of iron^50^,^51^ released from the melting winter sea ice transformed the glacial SIZ into a seasonally high productivity region governed by primary producers with low Si:N demand^5^, leading to enhanced utilization of nitrate^3^,^13^, slightly reduced consumption of silicic acid and low opal export^50^ (Fig. 4f). A similar productivity regime was triggered by iron-fertilization experiments in the modern SO, showing that involved diatoms (for example, *Chaetoceros*) follow a ‘boom-and-bust' life cycle strategy characterized by rapid biomass build-up during favourable growth conditions, succeeded by mass mortality and rapid population decline. Such a productivity regime results in enhanced organic carbon but low biogenic opal export to the deep ocean and thus leads to the decoupling of biogenic carbon and opal export^12^. In contrast to modern conditions, this would convert the glacial SIZ, which was enlarged during the glacial^34^,^52^, into an efficient carbon sink during spring–summer. Authigenic uranium concentrations, a potential proxy for organic carbon deposition, support this view, because they display increased values in sediments deposited in the glacial SIZ^50^.

For the area of the GWIE (PS1778-5), modelling suggests even longer-lasting spring–summer stratification compared with the glacial SIZ (PS1768-8) (Fig. 5). However, in this environment a different productivity-export regime developed. This regime is characterized by the production of thick-shelled diatoms (Si:N ratios >4, ref. 53) leading to enhanced silicic acid utilization (δ^30^Si_diat_=+1.62‰ to +1.86‰) at the sea surface and high percentages of biogenic opal in the sediments^5^,^50^ (Fig. 4h,j). Species involved (for example, *Fragilariopsis kerguelensis*)^5^ follow a ‘persistence' strategy and are hallmarked by enhanced ability to withstand grazing pressure. They are most prominent producers in the modern iron-limited open SO and present efficient silica sinkers^12^ with a major contribution to the modern Antarctic opal belt^5^. Sediment records suggest that this regime extended from the glacial SIZ into the area of the modern Subantarctic Zone as mirrored by increased opal flux and dominant deposition of *F. kerguelensis*^5^,^50^.

The northward displacement of the zone of enhanced opal burial (opal belt) together with winter sea-ice expansion^50^ during glacial periods was identified to represent a phenomenon most difficult to explain^3^. Queries concern the northward transfer of required nutrients from the glacial Antarctic Zone, assuming that surface water stratification resulted in reduced nutrient supply and almost complete consumption of available nutrients in this zone during the glacial^3^,^13^. Suggested explanations address increased leakage of silica into the Subantarctic region^3^,^54^ and northward-shift in wind-driven upwelling^13^. We emphasize a process (nutrient supply by winter mixing) that has been previously rejected^13^. Our modelling suggests that the shallow spring–summer stratification was disrupted by a significant MLD increase during glacial austral winter. The simulations indicate that the winter MLD is more than double the summer MLD in the area of PS1768-8 (∼200 m) and up to 350 m close to the GWIE (Fig. 5a). Around the GWIE and north of it, thus in the zone of enhanced glacial opal export^50^, modelling indicates even deeper ML conditions during glacial winter (Fig. 5). Such a deep MLD would allow for efficient nutrient refueling of glacial winter surface waters. A deep winter mixing would provide high nutrient (for example, Si(OH)_4_) availability at the onset of spring–summer production when surface water stratification is suggested to develop. A similar winter mixing process between ML, Winter Water and CDW is also suggested to take place in the modern SO (refs 29, 30). During spring–summer stratification, nutrient supply to the ML may be accomplished by diapycnal diffusion as described from the modern SO (ref. 55).

Amplification of the nutrient injection into the glacial surface ocean may stem from the presence of a deep reservoir that was more enriched in Si(OH)_4_ compared with modern conditions. The δ^30^Si_diat_ values at both studied sites point to similar isotopic compositions of the glacial ML and the modern ML (Fig. 3). However, the glacial radiolarian-derived δ^30^Si_Si(OH)4_ signal that can be related to surface–subsurface conditions (fraction >250 μm) is close to modern δ^30^Si_Si(OH)4_ values reported from the nutrient replete Winter Water and CDW in the Atlantic sector south of the Antarctic Polar Front. In contrast to the Holocene, the glacial radiolarian-derived δ^30^Si_Si(OH)4_ values from the fractions 125–250 μm and >125 μm, which reflect intermediate and deeper water conditions as well, are distinctly lower. These lower values fall in the range of δ^30^Si_Si(OH)4_ values reported from the modern deep Northwest Pacific (approximately +0.5‰), which are related to the highest Si(OH)_4_ concentrations observed in the World Ocean (∼160–180 μmol l^−1^)^20^ (Fig. 3b). Although our estimates still bear uncertainties, our reconstructions using different radiolarian fractionation offsets consistently result in lower glacial δ^30^Si_Si(OH)4_ values and thus higher glacial Si(OH)_4_ concentrations in SO intermediate deepwaters compared with the Holocene.

The suggested presence of higher Si(OH)_4_ concentrations in glacial circumpolar deep waters may be challenged by sponge spicule-based δ^30^Si records from the Scotia Sea, interpreted to indicate that the glacial deep Si(OH)_4_ concentrations were not different from modern conditions^21^. However, the records exhibit very negative δ^30^Si values (−3‰ to −3.5‰) and thus are in a range where the application of this proxy to sponge spicules is prone to larger uncertainties^56^. Another sponge spicule-derived δ^30^Si record from the area straddled by the Subtropical Front (ODP1089, Fig. 1), indicating no significant glacial–interglacial contrast in bottom water Si(OH)_4_ concentration in the Atlantic sector^22^, is not in conflict with our results. Indeed, this observation allows for approximation of the northern extent of higher Si(OH)_4_ concentrations trapped in the glacial SO. Presuming a similar relationship between modern and glacial biogenic opal deposition and decline of high silicic acid concentration throughout the water column (Supplementary Fig. 12), the northward displacement of the biogenic opal belt by ∼5° in latitude^50^ would place the glacial silicic acid front in the area between 50 and 45°S, but not as far north as the area of site ODP1089 (Fig. 1). This is in line with a northward migration of the Subantarctic Front as postulated from the mapping of ^14^C reservoir ages^57^. Further support comes from the pattern of LGM surface water temperature^34^ and model simulations, indicating a frontal northward displacement by ∼5°–7° in latitude from interglacial to glacial conditions (Supplementary Fig. 10).

A glacial SO trapping nutrients more efficiently than at present is consistent with the scenario of an Antarctic deep water body, whose age relative to the atmosphere was more than two times older than during the Holocene, and which was presumably CO_2_ enriched^57^ and characterized by increased salinity as mirrored by our model^23^ and suggested by proxy data^58^. Possible mechanisms that have an impact on the nutrient trapping may include wind-generated changes of upwelling and downwelling in the SO, sea-ice extent variability and the availability of iron^54^. A glacial northward export of the nutrient Si (Silicic Acid Leakage Hypothesis) would be in some conflict with a glacial SO nutrient enrichment, but data suggest that enhanced Si leakage was confined to the deglacial period^19^. Indeed, the sponge spicule-derived δ^30^Si record from the Subantarctic Atlantic (ODP1089, Fig. 1) indicates a bottom water spillout of the SO reservoir during this time, marked by a distinct δ^30^Si excursion towards more negative values (increased Si(OH)_4_ concentrations)^22^. Such Si(OH)_4_ export would support the hypothesis that an expansion of Si(OH)_4_-enriched Antarctic Bottom Water was the source for maximum opal fluxes (diatom blooms) in the coastal upwelling area off northwest Africa during the last deglaciation^59^.

The glacial–interglacial transition is characterized by successive changes, starting with the retreat of the sea ice that is accompanied by an increase in opal sedimentation between 18,000 and 16,000 years ago (Fig. 4e,f). This is followed by a steep increase in ocean ventilation at about 15,000 years ago^58^ (Fig. 4b), which marks the rapid intensification in Atlantic thermohaline circulation at the onset of the Bølling^60^. Assuming that the deglacial convergence between our δ^30^Si_diat_ and δ^30^Si_rad_ records reflects a deepening of the spring–summer MLD as proposed above, the MLD deepening would coincide with increased biogenic opal rain (Fig. 4d,f). Owing to a data gap in our >250 μm radiolarian record in PS1768-8, we cannot document exactly the onset of the change in surface water structure in the glacial SIZ. However, considering that the available record is closely tied to the retreat of sea ice and increasing deposition of biogenic opal (Fig. 4d–f), we speculate that the process of MLD deepening was initiated around 18,000 years ago, and that the MLD possibly reached its maximum thickness at around 14,000 years. In core PS1768-8, this is documented by the lowest δ^30^Si_diat_ values in the studied sediment interval (Fig. 4d). These δ^30^Si_diat_ minima that are only recorded by surface dwellers (diatoms) during a time of enhanced ventilation and maximum biogenic opal export suggest that the supply of silicic acid exceeded the consumption by diatoms during this time interval. This points to the injection of nutrient-rich deep waters into the euphotic zone^5^, interpreted to result from enhanced wind-driven upwelling governing biogenic opal production and export^6^.

The deglacial MLD deepening leads to conditions that persist in the Holocene (PS1768-8) as recorded by diatom and surface–subsurface radiolarian (>250 μm) δ^30^Si, which can be related to modern ML conditions in the Atlantic sector of the SO (Fig. 3a). The Holocene down-core data are comparable to available δ^30^Si_diat_ and δ^30^Si_rad_ data from surface sediments (assumed to reflect modern conditions) in the study area (Fig. 4d,h).

It has been postulated that the destratification and enhanced upwelling during the last deglaciation allowed for a CO_2_ release from the deep SO, providing a direct link to the coinciding increase in atmospheric CO_2_ (ref. 6). The primary mechanisms proposed to drive the destratification is a southward shift in the Southern Westerlies winds in response to a displacement of the Earth's thermal equator, the Intertropical Convergence Zone^6^. Another mode of operation can be derived from our sensitivity experiments applying a prescribed atmospheric CO_2_ increase from 180 to 240 p.p.m.v. (representing a surrogate for deglacial warming) and a poleward shift of the Southern Westerlies wind belt by 3° (Supplementary Figs 10 and 11). The sensitivity experiments show that the poleward shift of the Westerlies has a negligible effect on the position of the Antarctic Polar Front and MLD in comparison with the changes induced by the increase in atmospheric CO_2_ (Supplementary Figs 10 and 11). This suggests that the destratification during the last deglaciation can be primarily attributed to sea-ice margin retreat induced by atmospheric warming and an associated southward shift of the seasonal sea-ice melting zone.

The view that sea ice presents the major player in governing SO glacial surface-water structure and related ocean–atmosphere exchange, nutrient cycling and biological productivity and export regimes entrains major implications to be considered for the estimation of SO effects on the climate system. Quantification of the impact of physical and biological processes in the SO on the glacial carbon cycle requires consideration of seasonal variability in sea-ice extent and related seasonal and spatial variability in surface ocean mixing rates. Other factors to be taken into account are the development of specific productivity regimes making the SIZ an efficient carbon sink and the area north of the sea-ice edge a region that primarily affects the Si cycle, and the potential establishment of a nutrient-enriched deep SO reservoir.

## Methods

### Stratigraphy

To generate reliable age models for the studied cores, which lack continuous foraminiferal isotope records and carbonate-based AMS^14^C data series, we considered the strategy and stratigraphic data presented in a compilation of last glacial sea-surface temperature and sea-ice records from the Atlantic sector of the SO^38^. We have revised the correlation of 12 cores including PS1768-8 and PS1778-5 using 53 AMS^14^C dates from 8 cores (with 3 AMS^14^C dates available from PS1768-8) in addition to intercore correlation based on different parameters. The AMS^14^C dates were converted to calendar years and presented as cal. ka BP (10^3^ years before present). Parameters used for intercore correlation include the abundance pattern of the radiolarian *Cycladophora davisiana* and the diatom *E. antarctica*, together with foraminiferal oxygen isotope records, if available. Correlations were performed with AnalySeries 2.0 (ref. 61). The age assignment of the radiolarian and diatom abundance pattern was inferred from AMS^14^C dates obtained from 12 cores from the study area^38^. Stratigraphic pointers for both cores (including the AMS^14^C dates from PS1768-8) and their definition are presented in Supplementary Tables 5 and 6. Although this approach to construct age models for SO records bears uncertainties due to interpolations, especially in (mostly glacial) intervals that could not be dated by continuous ^14^C dates or δ^18^O data, we are confident that our age model is robust enough to allow for appropriate documentation of the environmental development from the last glacial into the present interglacial. According to our age models, the summer SST and WSIC began to shift towards Holocene values between 18 and 16 cal. ka BP (Fig. 4 and Supplementary Figs 7 and 8). This timing fits well with the onset of Southern Hemisphere warming and the start of CO_2_ release documented in Antarctic ice cores, dated independently of our approach^62^,^63^. Increase and decline of biogenic opal sedimentation is similar to the pattern recorded from the nearby core TN057-13 (ref. 6). However, in contrast to TN057-13, the biogenic opal, summer SST and WSIC records in our core PS1768-8 display no distinct variations, which can clearly be attributed to short-term climate variability (for example, the Antarctic Cold Reversal) during the last deglacial period. This may be attributed to three- to fourfold higher sedimentation rates at site TN057-13 (ref. 6) compared with site PS1768-8.

### Isotopes in biogenic opal

A prerequisite for measuring δ^18^O and δ^30^Si in diatom and radiolarian opal is the careful extraction and separation of sufficient purified material from both microfossil groups (Supplementary Figs 1–4). This is because the different life strategies and depth habitats of the two microfossil groups and different species within these groups, as well as contamination by sponge spicules and non-biogenic components (for example, rock fragments and clay minerals), may affect the isotope signal. For our study we have applied a new method, which allows for the separation of pure diatom and radiolarian fractions from the same sample aliquot. With our technical setup, an average of 2 mg purified diatom or radiolarian opal per sample is needed to obtain combined δ^18^O and δ^30^Si measurements from the same sample aliquot. This represents the lowest amount yet used for such combined measurements in comparison with other measuring techniques^64^. Considering that replicates and triplicates should be measured, as far as sample availability allows, to test the reproducibility of the measurements, the amount of opal to be separated and to be enriched increases accordingly. This precludes in general the separation of radiolarians by picking single radiolarian skeletons, because such a procedure takes an extraordinary expenditure of time. To generate well-established and large sample sets, a routine preparation method has been developed allowing for the removal of non-biogenic components and biogenic carbonate followed by the separation and enrichment of radiolarians and diatoms in specific size fractions.

Sample preparation includes wet-chemical cleaning and extraction of radiolarian and diatoms through different sieving and settling techniques (Supplementary Fig. 1). The samples are first washed with HCl and H_2_O_2_ to remove carbonates and organic material. Mineral grains are removed through density separations (on average ten density treatments for silica-rich sediments from the SO) using specific sodium polytungstate solutions. The separation of radiolarians from diatoms and sponge spicules is accomplished through several sieving and settling steps combined with ultrasonic treatment. Our tests show that after ultrasonic treatment radiolarians are still intact, whereas diatoms break up and can be removed through sieving. Repetitions of ultrasonic treatment and sieving steps lead to the separation and enrichment of radiolarians and diatoms in different size fractions. For isotope determinations, we use the pure 10–40 μm diatom fraction (both cores), the >250-μm and the 125- to 250-μm pure radiolarian fraction for core PS1768-8. As there was not enough material, we could only use one radiolarian fraction (>125 μm) for core PS1778-5 and one radiolarian fraction (>250 μm or >125 μm) for each surface sediment sample (Supplementary Tables 3 and 7). The smaller size fraction 40–125 μm contains radiolarians and diatoms of similar size, which are difficult to separate from each other and thus were not used for isotope measurements. Microscopic slides for determining the species composition of diatoms and radiolarians were prepared after completion of the preparation and separation of the preparation line (Supplementary Fig. 1).

For the combined oxygen and silicon isotope measurements, the cleaned radiolarian and diatom samples were dehydrated at 1,100 °C by inert gas flow dehydration under a He flow and further reacted to SiF_4_ and O_2_ by laser fluorination under BrF_5_ atmosphere. The liberated oxygen was cleaned of any byproducts and analysed with a PDZ Europa 2020 mass spectrometer according to the method described in refs 65, 66. The ^18^O/^16^O reference ratio of known isotopic composition is measured in analogy to the ^18^O/^16^O sample and the final δ^18^O value was calculated relative to Vienna standard mean ocean water. For silicon isotope measurements, the separated and cleaned SiF_4_ gas was directed into glass vials and measured separately with a Finnigan MAT 252 mass spectrometer and measured against the ^30^Si/^28^Si reference ratio of a SiF_4_ gas of known isotopic composition^18^. The final δ^30^Si value was calculated relative to NBS-28. To test the reproducibility of the measurements at least, replicates and triplicates were measured on all samples with sufficient amount of material (Supplementary Tables 3 and 7–12). The analytical precision of silicon isotope measurements was better than ±0.12‰ (δ^30^Si; 1*σ*) for all used working standard materials^18^. The overall precision for all working standards used for oxygen isotope measurements lies between±0.2‰ and 0.3‰ (1*σ*) (refs 40, 66).

### Purity of samples prepared for isotope measurements

In general, contamination of biogenic opal samples by minerals (for example, quartz, feldspar, micas, clay minerals, rock fragments and volcanic tephra) can bias, especially the δ^18^O signal towards lower values^17^,^40^, a pattern that may lead to misinterpretation of the isotope results. Considering that the contaminants may be silicates that are difficult to remove with the generally applied cleaning methods, the purity of the samples cleaned for isotope measurements needs to be tested. We can document that the cleaning procedure applied in our study leads to very well-purified diatom and radiolarian samples. Our testing of samples from core PS1768-8 using inductively coupled plasma optical emission spectrometry and energy-dispersive X-ray spectrometry indicates (1) a high degree of purification with the near absence of elemental compositions, indicating non-biogenic components, and (2) SiO_2_ contents ranging between 98.5% and 99.5% (inductively coupled plasma optical emission spectrometry), and 97.9% and 99.8% (energy-dispersive X-ray spectrometry) (Supplementary Table 1).

### Effect of dissolution on the isotope signals

Only few studies concern the potential impact of diagenesis on the opal isotope signal, which come to opposing results concerning the effect of dissolution on the δ^30^Si signal^19^. The diatoms extracted in this study are mainly composed of heavily silicified diatoms such as *E. antarctica* and *T. lentiginosa*, and are not affected by dissolution (Supplementary Fig. 3). The (rare) occurrence of very well-preserved thinly silicified diatoms (for example, *Rhizosolenia* sp.) confirms the excellent preservation of the diatom assemblage (Supplementary Fig. 3). This also concerns the radiolarian fractions, which are mainly composed of large and heavily silicified specimens, which are well preserved, although they were treated in the ultrasonic bath for several hours (Supplementary Fig. 4).

### Species composition of diatom and radiolarian fractions

For the isotopic measurements, we used a pure diatom fraction (10–40 μm) for the representation of surface-water conditions. This fraction mainly consists of the species *E. antarctica* (glacial indicator) and *T. lentiginosa* making up between 70% and 98%, and 78% and 97% of the species composition in the extracted diatom fractions of PS1768-8 and PS1778-5, respectively (Supplementary Fig. 5). The amount of these species in the original diatom assemblages is on average only between 20% and 29%. The difference in diatom species composition between the original sample and the extracted fraction results from our techniques for purification and extraction of a specific size class allowing for the generation of samples containing the least possible number of species. The shift from *E. antarctica*-dominated fractions to fractions with increased *T. lentiginosa* abundances occurs abruptly between three sample depths and is unrelated to the more gradual change in isotope signal across the glacial–interglacial shift (Supplementary Fig. 5). Small-sized sea-ice-related diatoms (for example, *Fragilariopsis curta*) that are not or only rarely included in the 10–40 μm diatom fraction do not affect the isotope signal.

For the isotopic measurements on radiolarians, we used three radiolarian fractions to document surface–subsurface and intermediate deepwater conditions. In core PS1768-8, we measured the >250 μm fraction, which is composed of two radiolarian species: *A. antarctica*, which accounts for >90% of radiolarians in this fraction, and *S. glacialis* (adult forms), which ranges between 1% and 10% in this fraction. As different radiolarian species live in different water depths, we used data from a plankton study in the Atlantic sector of the SO (ref. 25), to get information about the depth habitat of radiolarians in the upper 1,000 m of the water column (Supplementary Fig. 13 and Supplementary Table 2). Both species occur in the upper 200–400 m with *S. glacialis* predominantly occurring in the upper 100 m and *A. antarctica* in the upper 300–400 m. As there was not enough material to separate the >250-μm fraction from core PS1778-5, we used here the >125-μm fraction for radiolarian isotope measurements. This fraction is also dominantly composed of the two species *A. antarctica* and *S. glacialis*, but in contrast to the >250-μm fraction from core PS1768-8 the >125-μm fraction also contains deeper living species (for example, *Spongopyle osculosa*, *Spongogurus pylomaticus* and *Cromyecheinus antarctica*) that occur in water depths >400 m (ref. 25). As the δ^30^Si_rad_ values of the >125-μm fraction exhibit distinctly lower values (glacial average −1.06‰) than the δ^30^Si_rad_ values from the >250-μm fraction from core PS1768-8 (average glacial values +0.54‰), we segregated the 125- to 250-μm fraction from core PS1768-8 to get more information about this latitudinal difference in the δ^30^Si signature. The δ^30^Si_rad_ measurements from this fraction show distinctly lower values (glacial average −0.67‰) than the δ^30^Si_rad_ values from the >250-μm fraction. The fraction 125–250 μm also includes deeper dwelling radiolarians similar as in the >125-μm fraction from core PS1778-5. This suggests that the presence of deeper dwelling radiolarians shifts the δ^30^Si signal to lower values. *C. davisiana*, a species typical for glacial assemblages, is not included to the fractions >125 μm because of its small size, which impedes a potential impact of this species on glacial results.

### Estimation of δ^30^Si_Si(OH)4_ and Si(OH)_4_ concentrations

The estimation of Holocene and glacial silicic acid changes were carried out as follows:

1. The average δ^30^Si values of the diatom (10–40 μm) and radiolarian fractions (>250, 125–250 and >125 μm) for the Holocene (until 12 cal. ka BP) and the last glacial period (∼19–29 cal. ka BP) were calculated (Supplementary Table 4).

2. Based on the average diatom and radiolarian δ^30^Si values, the δ^30^Si_Si(OH)4_ values for the Holocene and last glacial period were calculated using the formula:


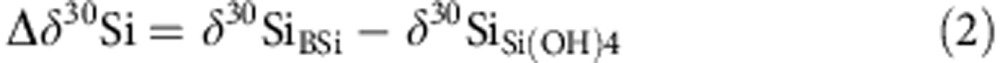



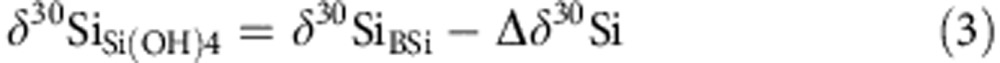


where δ^30^Si_BSi_ is the silicon isotope composition of biogenic silica (diatoms or radiolarians), δ^30^Si_Si(OH)4_ is the silicon isotope composition of the input sea water (Supplementary Table 4) and Δ*δ*^30^Si is the fractionation offset. The calculations were performed using different Δδ^30^Si values: Δδ^30^Si_diat_=−1.1‰ (ref. 24), Δδ^30^Si_rad_=−0.8‰ (average Δδ^30^Si_rad_ this study, Supplementary Table 4), Δδ^30^Si_rad_=−1.2‰ (average Δδ^30^Si_rad_ of this study and ref. 32), Δδ^30^Si_rad_=−1.5‰ (average Δδ^30^Si_rad_ of ref. 32).

3. The reconstructed δ^30^Si_Si(OH)4_ values were related to δ^30^Si_Si(OH)4_ values and Si(OH)_4_ concentrations from different water masses reported from modern water-column studies^20^,^28^,^29^. We note that our reconstructed δ^30^Si_Si(OH)4_ values reflect a rather broad range in Si(OH)_4_ concentrations (Fig. 3 and Supplementary Table 4). In spite of this large range in variability, our calculated δ^30^Si_Si(OH)4_ values for the diatoms and different radiolarian fractions are in the range of δ^30^Si_Si(OH)4_ values of specific water masses and reflect their range in Si(OH)_4_ concentrations^20^,^28^,^29^ (Fig. 3 and Supplementary Table 4).

### Reconstruction of WSIC

WSIC (%) was estimated from the diatom assemblage composition preserved in the sediment records using the transfer function technique. For our study we selected the estimations obtained with the Imbrie and Kipp transfer function method using a setup with 172 reference sites, 28 diatom taxa/taxa groups, logarithmic-transformed diatom data, quadratic regression and a three-factor model with a root mean square error of prediction of 7.3% (ref. 35). Similar patterns of sea-ice concentration were also obtained with three other transfer function techniques, all showing the onset of sea-ice retreat after the last glacial period at around 18,000 years ago^35^. Although the diatom signal used for the estimate of WSIC is based on a signal produced during the diatom spring–summer bloom, experimental (sediment trap) data and statistical test show that this signal reflects the occurrence probability of winter sea ice, which is well correlated with the WSIC at a given site (ref. 35 and references included therein).

### Model setup and experimental design

For our simulations we use the experimental settings of the LGMW simulations in ref. 23, which is integrated for 4,000 years from a cold ocean state, to evaluate MLD characteristics under LGM (21 ka) and present-day conditions, as well as for the sensitivity experiments. The thermodynamics of sea ice relate changes in sea-ice thickness to a balance of radiant, turbulent and oceanic heat fluxes. The effect of snow accumulation is taken into account, along with snow–ice transformation when the snow–ice interface sinks below the sea level because of snow loading. The impact of ice growth and ice melting is included in the model, assuming a sea-ice salinity of 5 PSU^46^. In experiment WIND, the implementation of the poleward wind field shift (3° southwards) of the Westerlies in the SO (experiment WIND, Supplementary Figs 10 and 11) has been performed in analogy to ref. 43. In experiment CO2, a deglacial CO_2_ increase from 180 to 240 p.p.m.v. has been applied. Both experiments have been integrated for 600 years. All figures show climatological mean characteristics averaged over a period of 100 years at the end of each simulation.

## Additional information

**How to cite this article**: Abelmann, A. *et al*. The seasonal sea-ice zone in the glacial Southern Ocean as a carbon sink. *Nat. Commun.* 6:8136 doi: 10.1038/ncomms9136 (2015).

## Supplementary Material

Supplementary Information Supplementary Figures 1-13, Supplementary Tables 1-12 and Supplementary References
