# Corrigendum: The seasonal sea-ice zone in the glacial Southern Ocean as a carbon sink

**DOI:** 10.1038/ncomms12004

**Published:** 2016-06-15

**Authors:** Andrea Abelmann, Rainer Gersonde, Gregor Knorr, Xu Zhang, Bernhard Chapligin, Edith Maier, Oliver Esper, Hans Friedrichsen, Gerrit Lohmann, Hanno Meyer, Ralf Tiedemann

*Nature Communications* 6:8136 doi: 10.1038/ncomms9136 (2015); Published 09182015; Updated 06152016

The original version of this Article failed to fully credit the use of the Ocean Data View software in figure 1 and supplementary figure 12, which appears below:

Schlitzer, R., Ocean Data View, http://odv.awi.de, 2016.

