# Supplementary material for: The seasonal sea-ice zone in the glacial Southern Ocean as a carbon sink
Source: Nat Commun. 2015 Sep 18;6:8136. doi: 10.1038/ncomms9136 (PMC4595604; doi:10.1038/ncomms9136)
Supplement: Supplementary Information — Supplementary Figures 1-13, Supplementary Tables 1-12 and Supplementary References [file ncomms9136-s1.pdf]

SUPPLEMENTARY INFORMATION

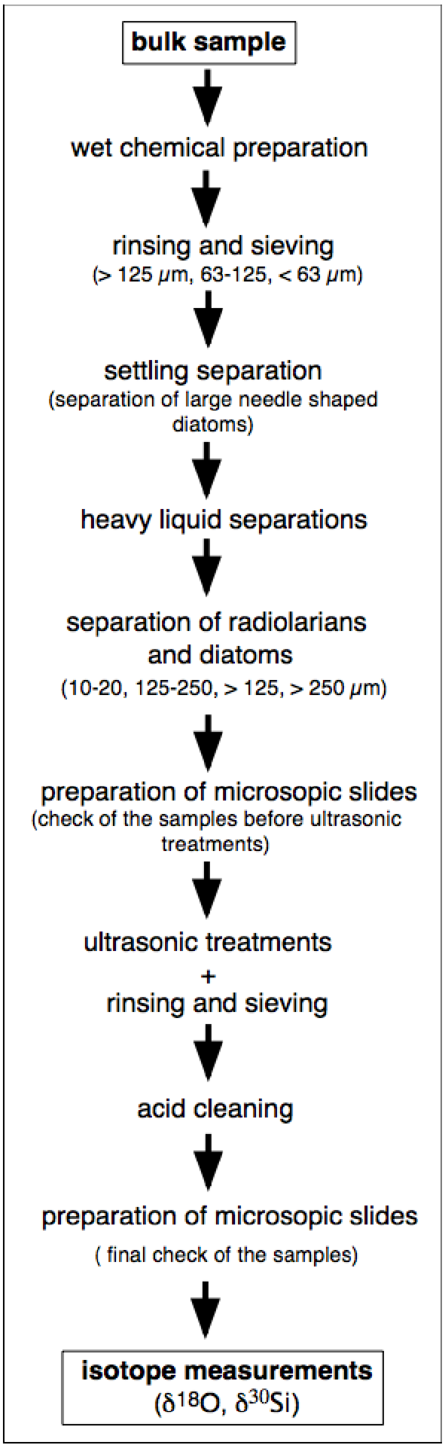

Supplementary Figure 1. Preparation line for purifying and separating diatom and radiolarian opal for isotope measurements.

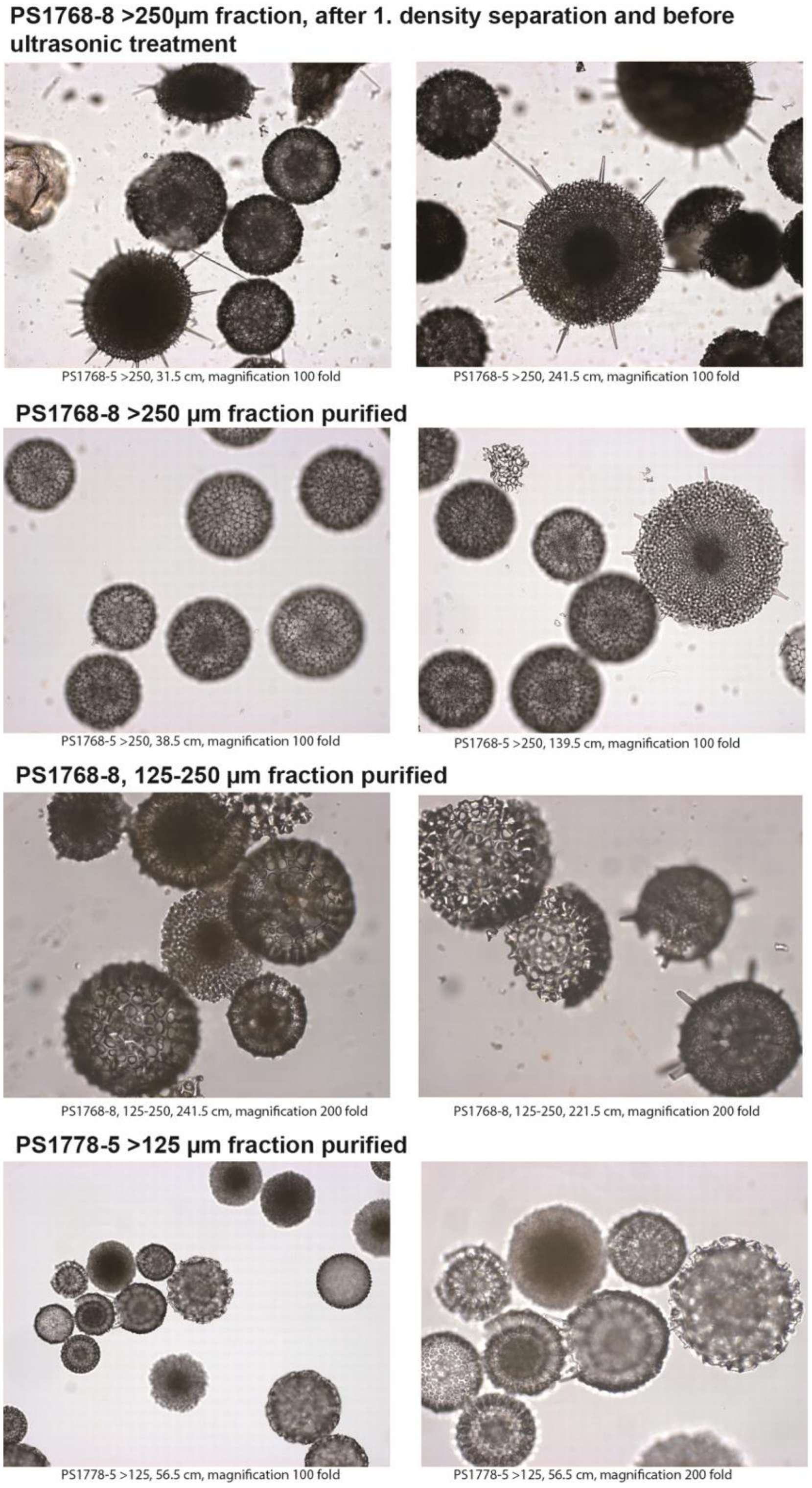

Supplementary Figure 2. Examples of different size class fractions of radiolarians obtained through several sieving steps.

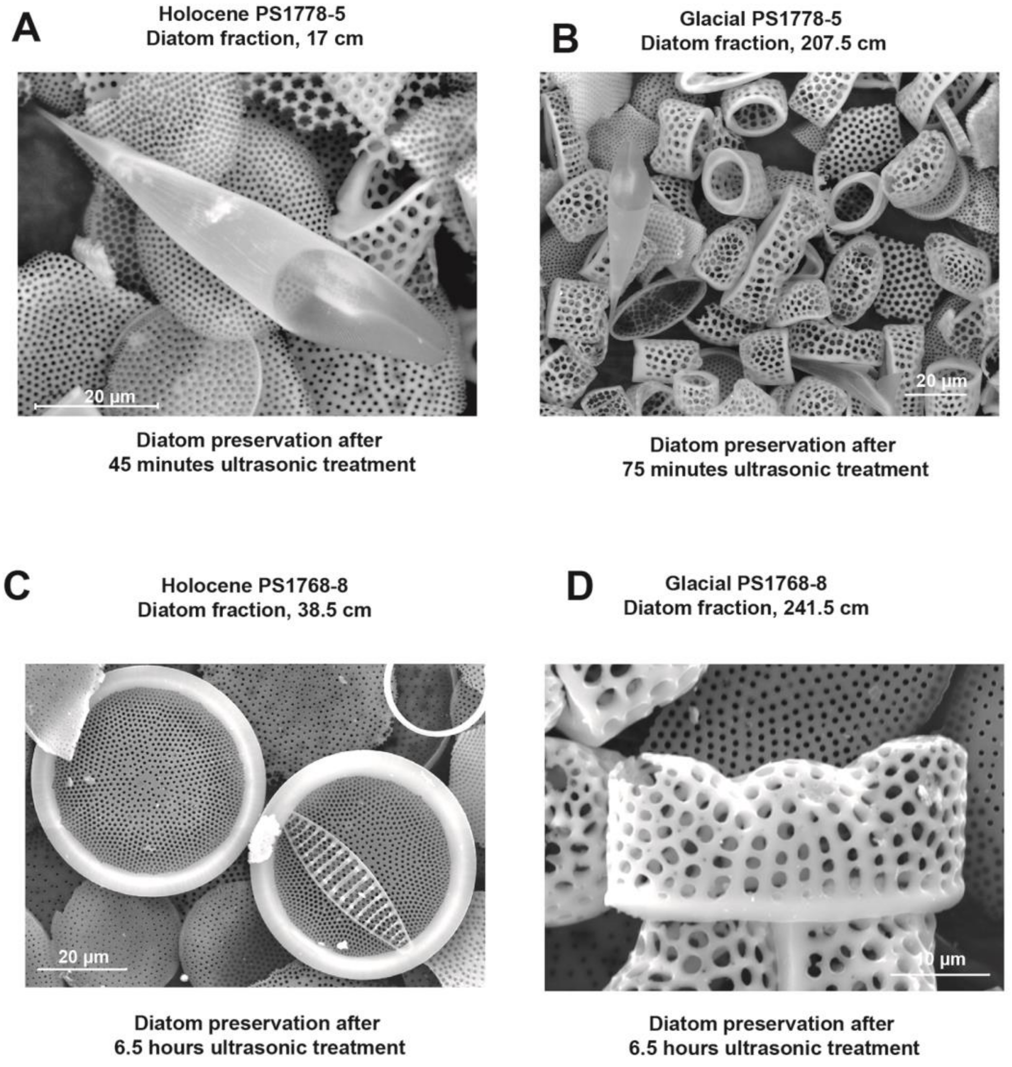

Supplementary Figure 3. Scanning Electron Microscope (SEM) images of pure diatom fractions. (A,B) Images from Holocene and glacial diatom fractions from core PS1778-5. The (rare) occurrence of thinly silicified but very well preserved diatom *Rhizosolenia antarctica* together with centric diatoms **(A)** and *Eucampia antarctica* **(B)** indicates excellent preservation of the purified diatom assemblages. **(C, D)** Images from Holocene and glacial diatom fractions from core PS1768-8. Although the samples were treated 6.5 hours in the ultrasonic bath the presevation of the diatoms is very good. **(C)** *Thalassiosira oliverana* and *Fragilariopsis kerguelensis* (rare occurrence), **(D)** *E. antarctica*.

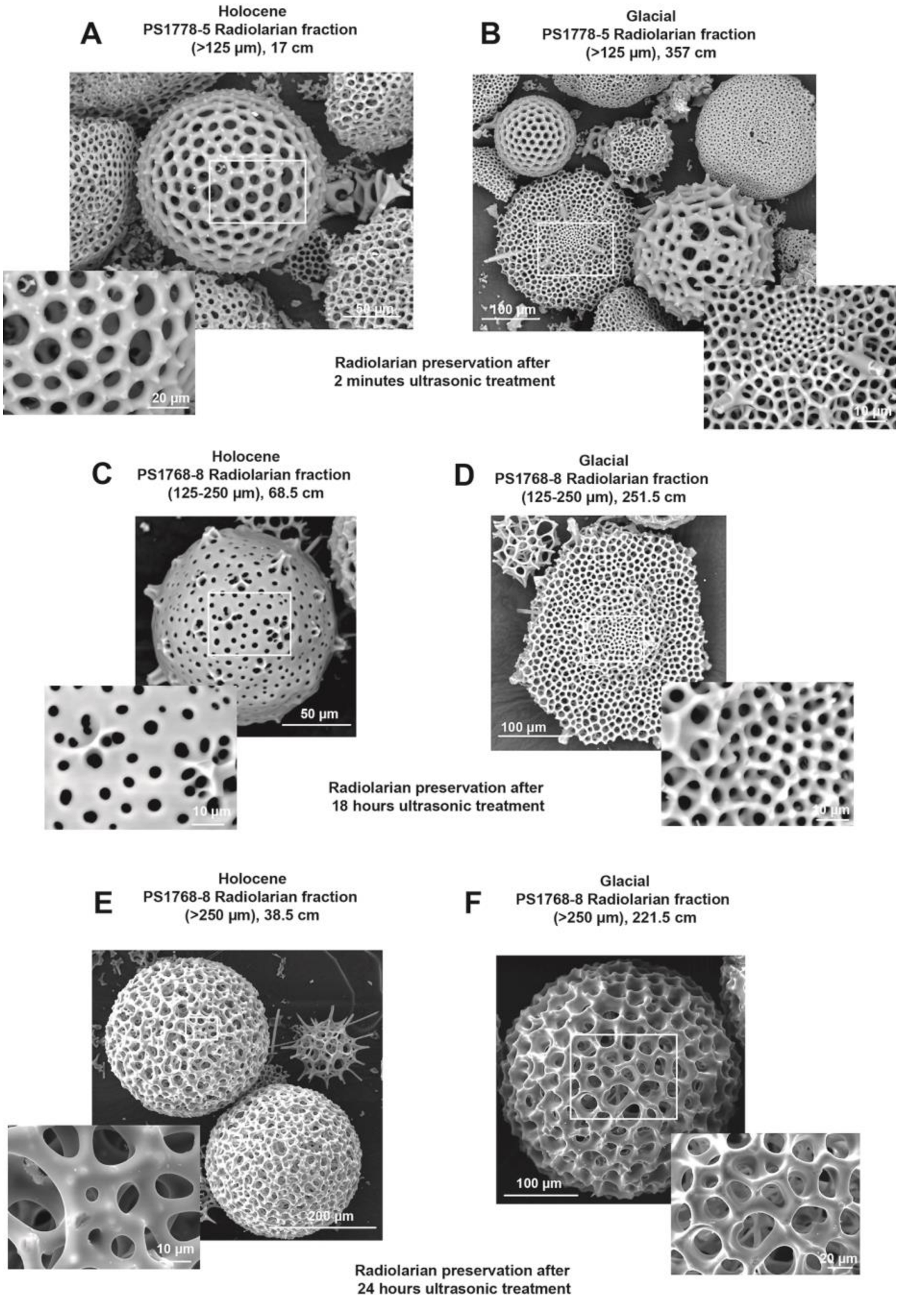

Supplementary Figure 4. Scanning Electron Microscope (SEM) images of pure radiolarian fractions. (A, B) Images from Holocene and glacial radiolarian fractions (>125 µm) from core PS1778-5. **(C, D)** Images from Holocene and glacial radiolarian fractions (125-250 µm) from core PS1768-8 after 18 hours ultrasonic treatment. **(E, F)** Images from Holocene and glacial radiolarian fractions (> 250 µm) from core PS1768-8 after 24 hours utrasonic treatment. Although the samples were treated for hours in the ultrasonic bath the presevation of the radiolarians in both cores is very good.

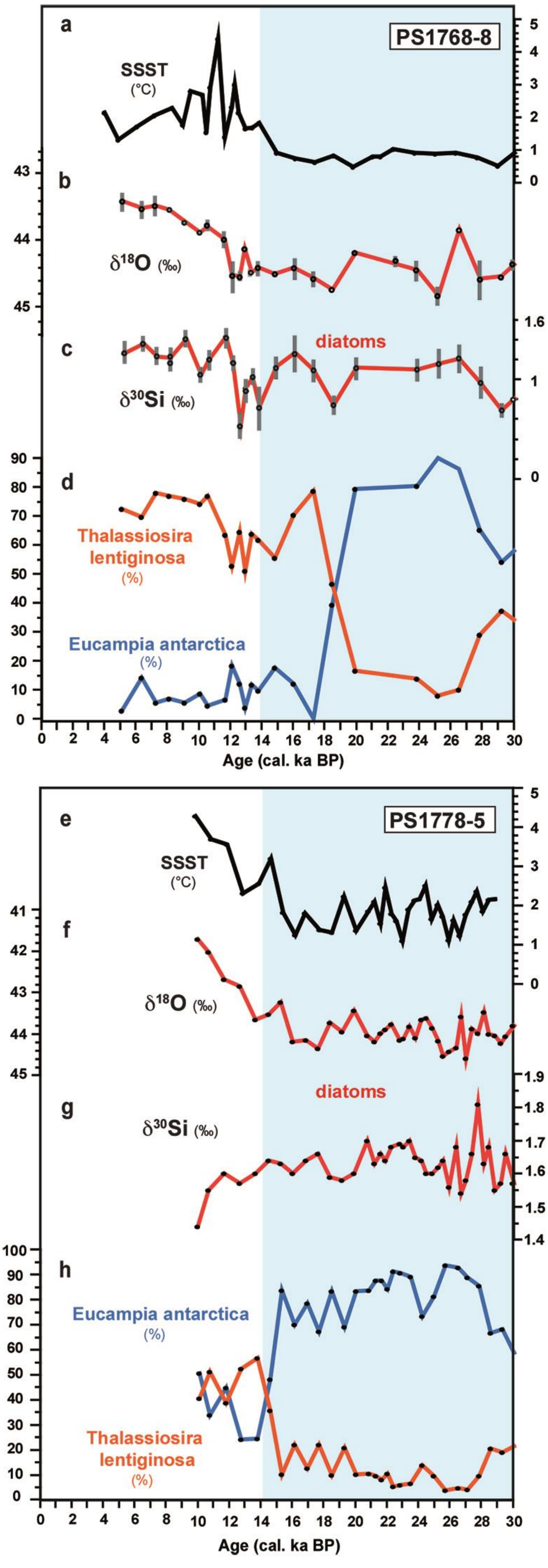

PS1768-8
a
SSST (°C)
b
δ18O (‰)
c
diatoms
δ30Si (‰)
d
Thalassiosira lentiginosa (%)
Eucampia antarctica (%)
Age (cal. ka BP)
PS1778-5
e
SSST (°C)
f
δ18O (‰)
g
diatoms
δ30Si (‰)
h
Eucampia antarctica (%)
Thalassiosira lentiginosa (%)
Age (cal. ka BP)

Supplementary Figure 5. Variations of the two most abundant diatom species in the diatom fractions used for isotopic measurements. The abundance variations in cores PS1768-8 and PS1778-5 are compared to $\delta^{18}O$ and $\delta^{30}Si$ measurements and summer sea surface temperature (SSST) changes. **(a-d)** Core PS1768-8 located in the GSIZ. **(a)** Diatom transfer-function-based estimates of summer sea-surface temperature (SSST) estimated from diatom assemblage compositions using the Imbrie and Kipp Method (IKM). The transfer function relies on a set up with 336 reference sites, 29 diatom taxa/taxa groups, logarithmic-transformed diatom data, quadratic regression and a three-factor model, which results in root mean square errors of prediction (RMSEP) of 0.8°C (ref. S1). **(b)** $\delta^{18}O$ and **(c)** $\delta^{30}Si$ records measured at the diatom fraction. **(d)** Relative abundances of the diatom species *Thalassiosira lentiginosa* and *Eucampia antarctica* in the diatom fraction extracted for isotope measurements. **(e-h)** Core PS1778-5 located close to the glacial winter sea-ice edge. **(e)** SSST estimations based on diatom IKM transfer function[S1]. **(f)** $\delta^{18}O$ and **(g)** $\delta^{30}Si$ records measured at the diatom fraction. **(h)** Relative abundances of the diatom species *T. lentiginosa* and *E. antarctica* in the diatom fraction extracted for isotope measurements. Blue-shaded area delineates MIS2 and the late part of MIS 3.

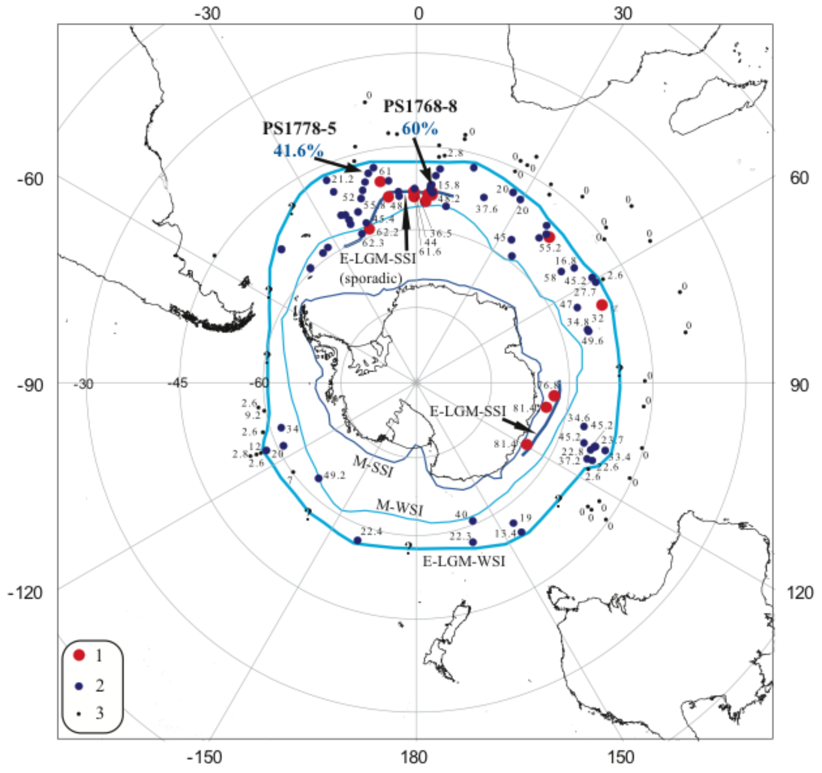

Supplementary Figure 6. Southern Ocean sea-ice distribution as reconstructed for the EPILOG-LGM time slice. Sea-ice distribution from ref. 34 including newly reconstructed winter sea-ice concentration (%) from cores PS1768-8 and PS1778-5 averaged over the EPILOG-LGM time slice (23.000-19.000 cal. yr BP). Signature legend: (1) concomitant occurrence of cold-water indicator *Fragilariopsis obliquecostata* (>1% of diatom assemblage) and summer sea ice concentration >0% interpreted to represent (sporadic) occurrence of summer sea ice; (2) presence of winter sea ice based on diatom sea ice indicators and transfer function derived winter sea-ice concentration estimates (values); (3) no sea ice. The modern maximum winter sea ice (M-WSI) is placed at the 15% September concentration according to ref. 67 and the EPILOG-LGM maximum winter sea-ice extent (E-LGM-WSI) follows this definition. This compilation shows that during the glacial site PS1768-8 was located in the northern seasonal sea-ice zone and site PS1778-5 was in the area of the sea-ice edge, where sea-ice concentration drops abruptly.

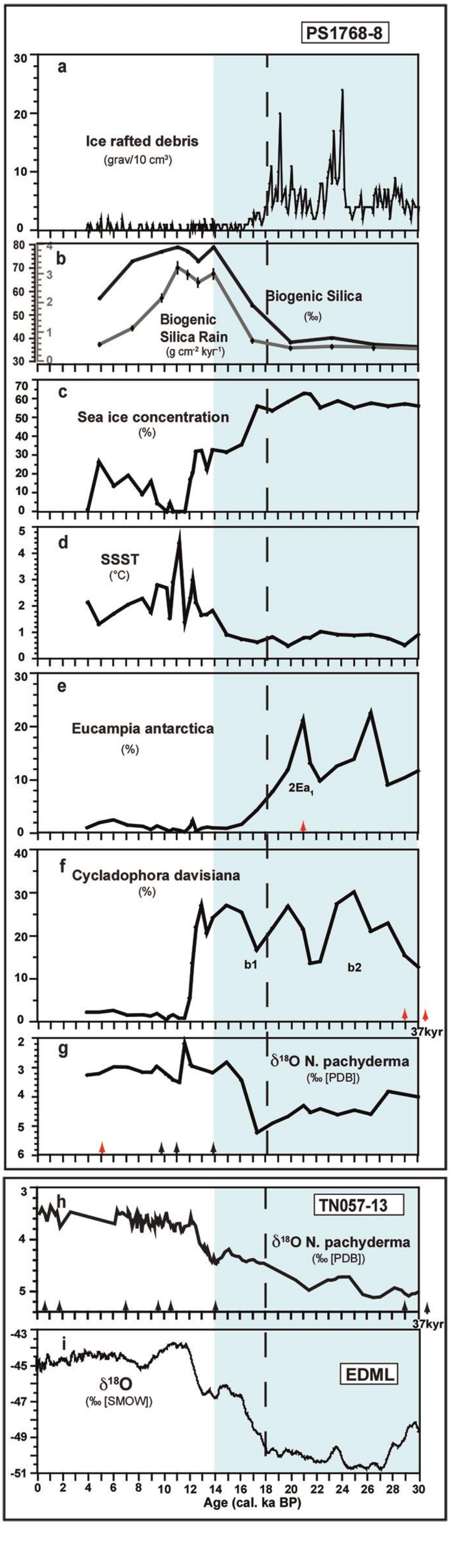

PS1768-8
a
Ice rafted debris
(grav/10 cm³)
b
Biogenic Silica
(‰)
Biogenic
Silica Rain
(g cm⁻² kyr⁻¹)
c
Sea ice concentration
(%)
d
SSST
(°C)
e
Eucampia antarctica
(%)
2Ea1
f
Cycladophora davisiana
(%)
b1
b2
37kyr
g
δ18O N. pachyderma
(‰ [PDB])
TN057-13
h
δ18O N. pachyderma
(‰ [PDB])
37kyr
i
EDML
δ18O
(‰ [SMOW])
Age (cal. ka BP)

Supplementary Figure 7. Proxies of core PS1768-8 used for dating and paleoceanographic interpretation compared to core TN057-13 and the EDML ice core. **(a-g)** Parameters of core PS1768-8: **(a)** Ice rafted debris (IRD) derived from the abundance of gravel clasts >2 mm per 10 cm^3, estimated from x-radiograph[S2]. **(b)** Biogenic silica percentages and biogenic silica rain rates[50]. **(c)** Diatom transfer function-based estimates of winter sea-ice concentration[35]. **(d))** SSST estimations based on diatom IKM transfer function[S1]. **(e)** Relative abundances of the diatom *Eucampia antarctica*[38] **(f)** Relative abundances of the radiolarian *Cycladophora davisiana*[5]. **(g)** $\delta^{18}O$ record of *Neogloboquadrina pachyderma* (sinistral)**[S3]**. **(h)** $\delta^{18}O$ record of *N. pachyderma* (sinistral) from core TN057-13 (ref. 15). **(i)** $\delta^{18}O_{H2O}$ record from EDML (EPICA Dronning Maud Land) ice core[69] against AICC2012 (Antarctic Ice Core Chronology 2012)[62]. Blue-shaded area delineates MIS2 and the late part of MIS 3. Vertical black dashed line marks the onset of warming seen in East Antarctic ice cores[69,S4]. Arrows in panels **e**, **f**, **g** and **h** indicate age pointer (black arrows mark AMS ^{14}C dates[15,38], red arrows mark ages obtained by diatom and radiolarian stratigraphy, and by $^{230}Th_{ex}$ constant flux modeling, see Supplementary Table 5). Foraminiferal-based oxygen isotope records from Quaternary records south of the Subantarctic Front generally rely on the surface-subsurface dwelling planktic foraminifer *N. pachyderma* (sinistral). Although obtained data may result in stratigraphically useful records[15,S5 ,S6], *N. pachyderma* (sinistral) oxygen isotope records may also display high amplitude anomalies during glacials and glacial-interglacial transitions whose nature remains difficult to be explained. Some of this may stem from contamination related to the very low carbonate record[S7], but the signals were also interpreted to be influenced by glacial meltwater supply[S8]. In PS168-8 **(g)** and PS1778-5 (Supplementary Fig. 8f) the $\delta^{18}O$ records of *N. pachyderma* (sinistral) display high amplitude signal shifts, e.g. between 18.000 and 16.000 cal. yr BP, thus are distinct from the diatom and radiolarian $\delta^{18}O$ records (Fig. 3c,g). Interestingly, the diatom and radiolarian $\delta^{18}O$

records are similar to a *N. pachyderma* (sinistral) record reported from core TN057-13 (ref. 15) **(h)**.

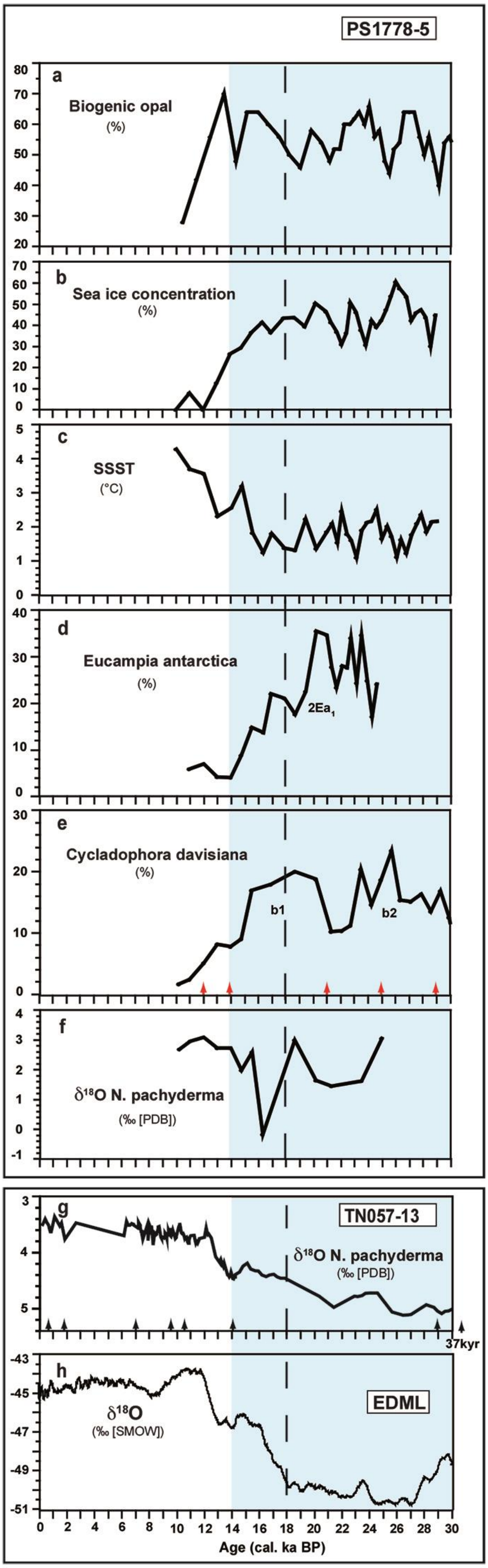

PS1778-5
a
Biogenic opal
(%)
b
Sea ice concentration
(%)
c
SSST
(°C)
d
Eucampia antarctica
(%)
2Ea1
e
Cycladophora davisiana
(%)
b1
b2
f
δ18O N. pachyderma
(‰ [PDB])
g
TN057-13
δ18O N. pachyderma
(‰ [PDB])
37kyr
h
EDML
δ18O
(‰ [SMOW])
Age (cal. ka BP)

Supplementary Figure 8. Proxies of core PS1778-5 used for dating and paleoceanographic interpretation compared to core TN057-13 and the EDML ice core. **(a-f)** Parameters of Core PS1778-5: **(a)** Biogenic silica percentages, **(b)** Transfer function-based estimates of winter sea-ice concentration[35]. **(c)** Transfer-function based estimates of summer sea-surface temperature (SSST)[S1]. **(d)** Relative abundances of the diatom *Eucampia antarctica*[38]. **(e)** Relative abundances of the radiolarian *Cycladophora davisiana*[S9]. **(f)** $\delta^{18}O$ record of *N. pachyderma* (sinistral). **(g)** $\delta^{18}O$ record of *N. pachyderma* (sinistral) from core TN057-13 (ref. 15). **(h)** δ^{18} record from EDML (EPICA Dronning Maud Land) ice core[69] against AICC2012 (Antarctic Ice Core Chronology 2012)[62]. Blue-shaded area delineates MIS2 and the late part of MIS 3. Vertical black dashed line marks the onset of warming seen in East Antarctic ice cores[69,S4]. Arrows in panels **e** and **g** indicate age pointer (black arrows mark AMS^{14}C dates[15], red arrows mark ages obtained by diatom and radiolarian stratigraphy, see Supplementary Table 6).

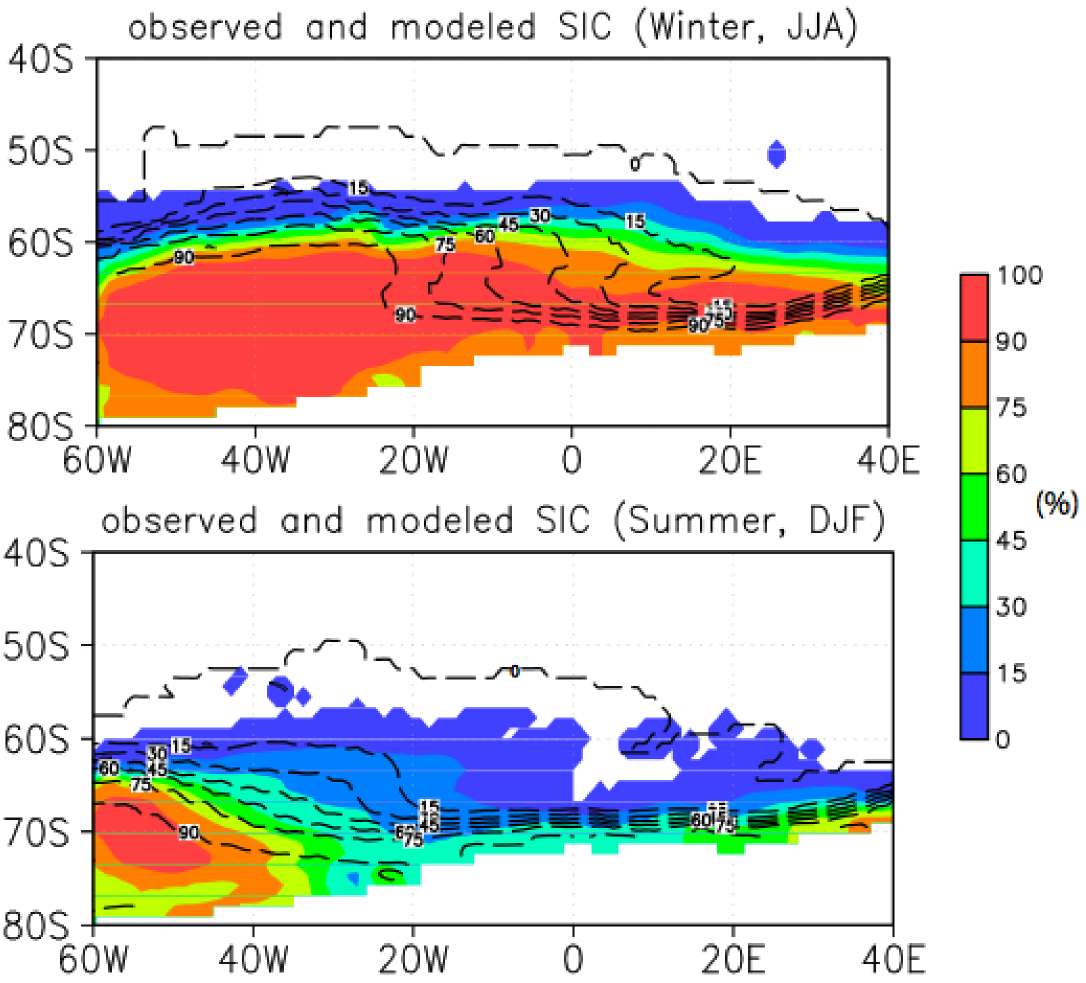

Supplementary Figure 9. Present day climatological mean sea ice concentration in the Atlantic sector of the Southern Ocean. Shown is the reconstructed average sea ice concentration between A.D. 1966-2000 (color-shaded area) derived from ref. S10, overlaid with contours of the climatological sea ice distribution as modeled by our AOGCM approach (contour lines).

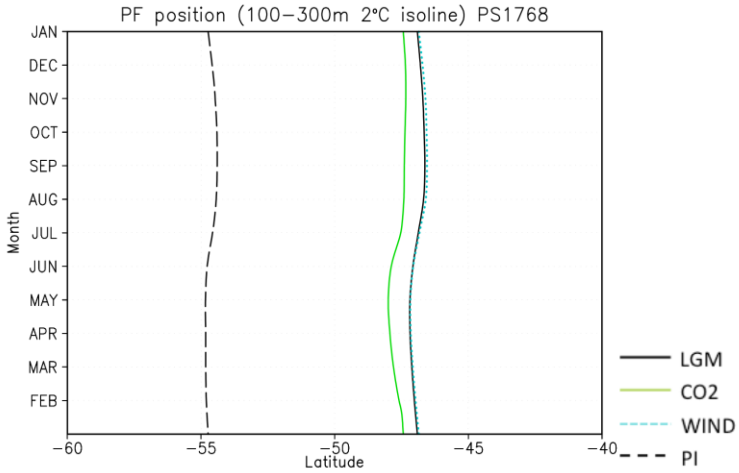

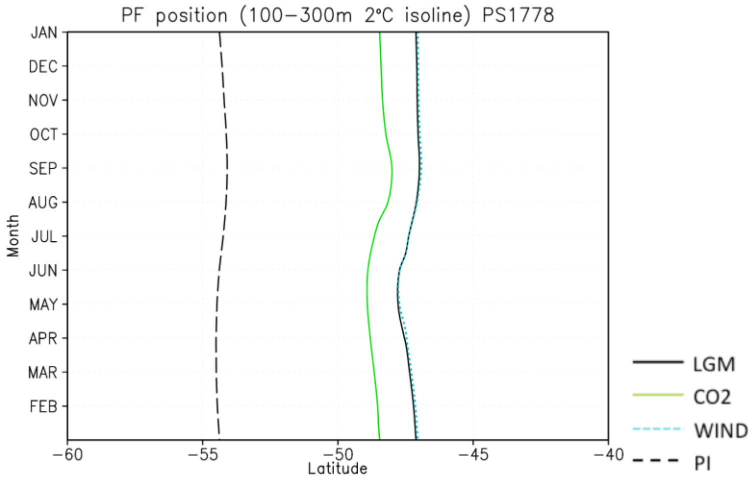

Supplementary Figure 10. Position of the Antarctic Polar Front (PF) in the deglacial sensitivity experiments. Displayed are the relative impact of a deglacial CO_2 increase from 180 ppmv to 240 ppmv (referred to as experiment CO2) and a poleward shift of the Westerlies in the Southern Ocean by 3° in latitude (referred to as experiment WIND) on the annual cycle of the latitudinal position of the Antarctic PF at the longitudes of the core locations (upper panel PS1768-8 and lower panel PS1778-5). Additionally, the simulated pre-industrial (PI) and the LGM positions of the Antarctic PF are shown. The Antarctic PF position is defined by the 2°C-isoline of the averaged temperature between a water depth of 100-300 m. A comparison of the glacial (LGM) and interglacial (pre-industrial) position of the PF indicates a glacial northward shift of ca. 5-7°. Furthermore similar to the response in

sea-ice cover (Supplementary Fig.11), the comparison between experiments CO2 and WIND shows a relatively pronounced southward shift of the Antarctic PF in experiment CO2. This suggests that the greenhouse-gas induced southward shift of the sea ice-melting zone represents a key control on the deglacial southward migration of the Antarctic PF (see Supplementary Fig.11 and figure caption therein for further details).

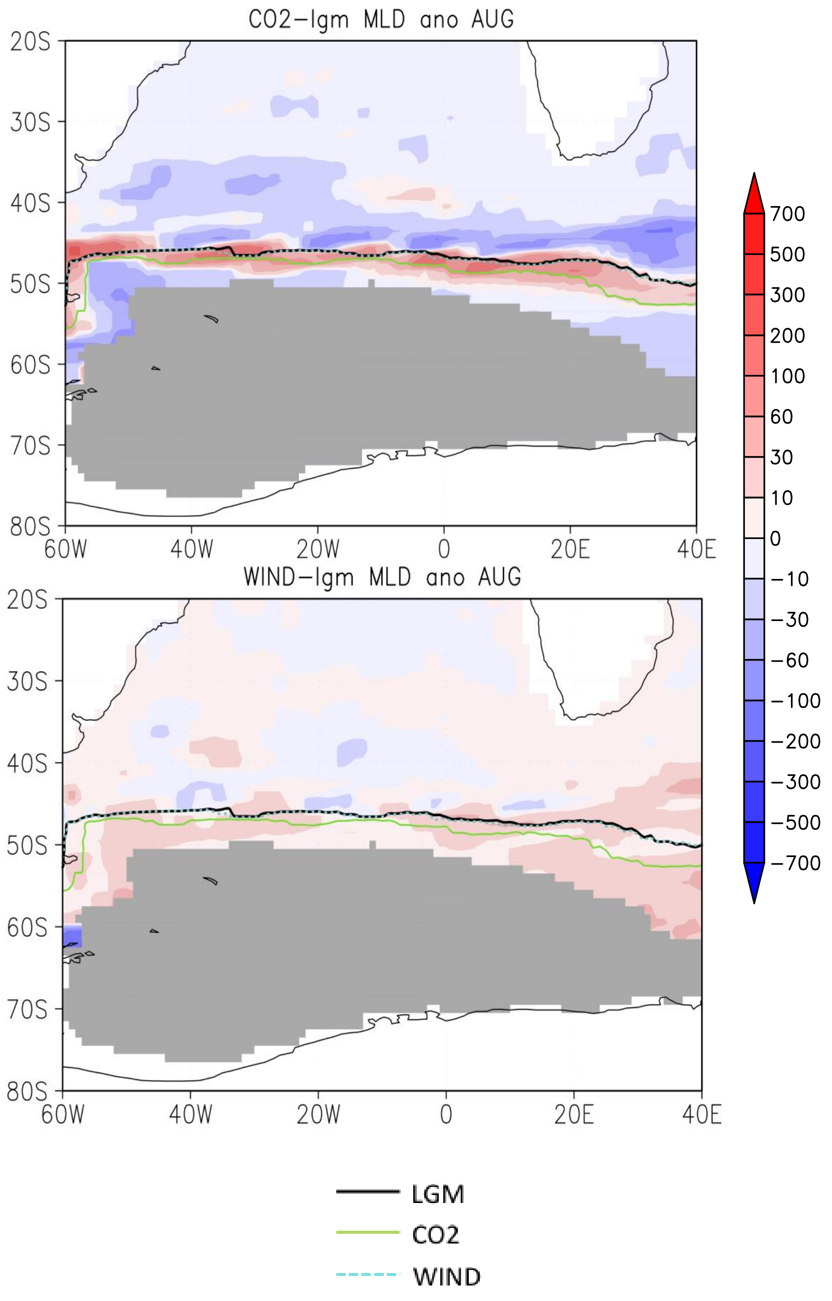

Supplementary Figure 11. Mixed layer depth changes and sea-ice concentrations in the deglacial sensitivity experiments. Displayed are the relative impacts on glacial MLD (m) of a deglacial CO_2 increase from 180 ppmv to 240 ppmv (referred to as experiment CO2, upper panel) and a poleward shift of the Westerlies in the Southern Ocean by 3° in latitude (referred to as experiment WIND, lower panel). The MLD changes are shown for the month August during Austral winter conditions. In both panels the MLD changes are overlaid with the same sea ice concentration (15%) of the corresponding month for the glacial state (black line), the WIND experiment (turquoise dotted line) and the CO2 experiment (green line). Areas with a sea-ice concentration of more than 90% in the glacial state are shaded in grey. The sensitivity experiments have been initialized with a glacial equilibrium simulation (simulation LGMW in ref. 23) and integrated for 600 years. The last 100 years of model output have been averaged and are shown as climatological mean values. In both experiments a deepening of the MLD is detected. A comparison between the two sensitivity experiments shows a relatively pronounced sea ice retreat and deeper MLD in experiment CO2. In this experiment the greenhouse-gas induced warming causes the sea ice retreat (region between the black to the green line), which is accompanied by a southward shift of the sea ice melting zone and deeper MLD in this region. A similar southward shift is also detected in the APF as shown in Supplementary Fig.10. Alternative scenarios to the greenhouse-gas induced warming in experiment CO2, might relate deglacial warming in the Southern Ocean e.g. to insolation changes or changes in the Atlantic meridional overturning circulation and associated inter-hemispheric heat transport.

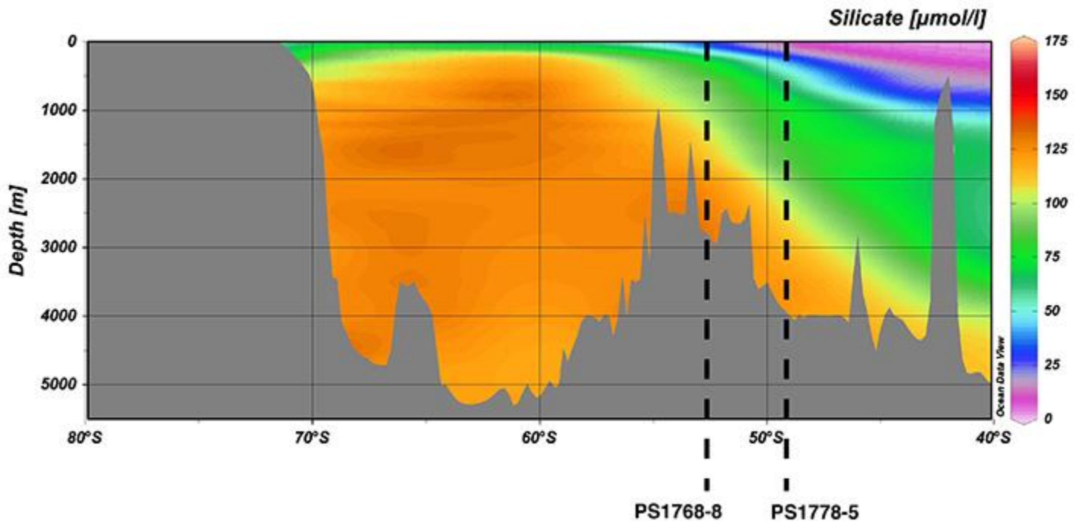

Supplementary Figure 12. Meridional depth sections of $Si(OH)_4$ concentrations at 0°. Stippled black lines indicate the locations of cores PS1768-8 and PS1778-5. The nutrient data are from ref. 33 and for imaging we used Ocean Data View[S11].

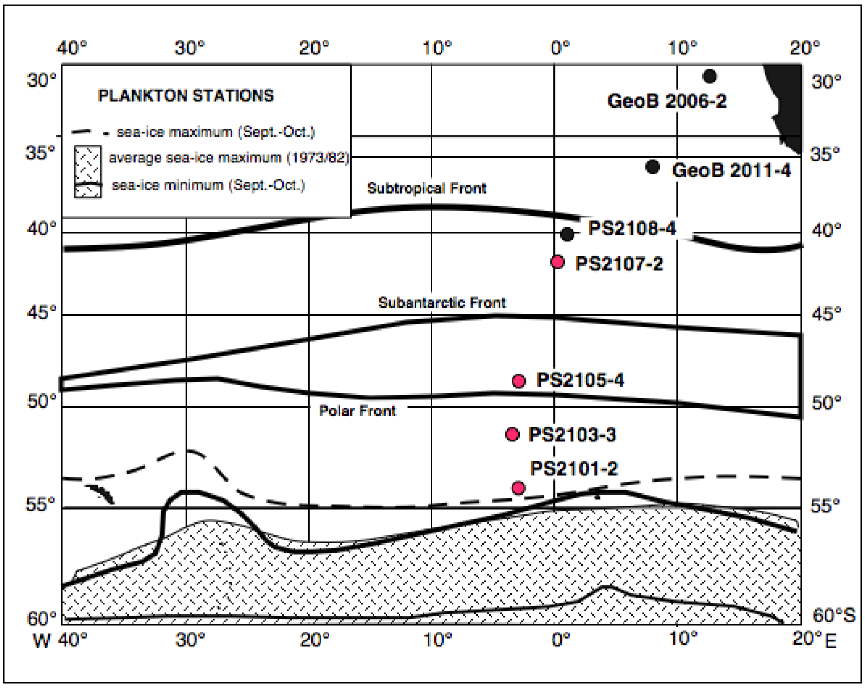

Supplementary Figure 13. Location of plankton stations used for determination of radiolarian depth habitats. For data see Supplementary Table 2 (from ref. 25).

Element	Depth (cm)	Radiolarians 31.5	68.5	191.5	241.5	Diatoms 31.5	68.5	191.5	241.5
ICP-OES									
Al_2O_3	%	0.0	0.1	0.1	0.0	0.0	0.0	0.5	0.6
CaO	%	0.1	0.1	0.1	0.1	0.0	0.0	0.1	0.1
Fe_2O_3	%	0.0	0.0	0.0	0.0	0.0	0.0	0.1	0.1
K_2O	%	0.1	0.1	0.1	0.1	0.1	0.1	0.1	0.1
MgO	%	0.0	0.0	0.0	0.0	0.0	0.0	0.0	0.1
MnO	%	0.0	0.0	0.0	0.0	0.0	0.0	0.0	0.0
Na_2O	%	0.6	0.7	0.7	0.6	0.3	0.4	0.5	0.5
P_2O_5	%	0.0	0.0	0.0	0.0	0.0	0.0	0.0	0.0
TiO_2	%	0.0	0.0	0.0	0.0	0.0	0.0	0.0	0.0
SUM	%	0.8	1.0	0.9	0.8	0.5	0.6	1.3	1.5
SiO_2	%	**99.2**	**99.0**	**99.1**	**99.2**	**99.5**	**99.4**	**98.7**	**98.5**
EDS									
Al_2O_3	%	0.0	0.0	0.0	0.0	0.0	0.0	0.1	0.4
CaO	%	0.1	0.1	0.1	0.1	0.0	0.0	0.1	0.0
Fe_2O_3	%	0.0	0.1	0.1	0.1	0.0	0.0	0.0	0.2
K_2O	%	0.1	0.1	0.1	0.1	0.0	0.0	0.1	0.1
MgO	%	0.1	0.1	0.1	0.0	0.1	0.1	0.2	0.1
MnO	%	0.0	0.0	0.0	0.0	0.0	0.0	0.1	0.0
Na_2O	%	0.6	0.7	0.6	0.8	0.0	0.0	0.0	0.8
Cl	%	0.3	0.3	0.2	1.0	0.0	0.0	0.3	0.0
SUM		1.1	1.3	1.1	2.1	0.2	0.2	0.9	1.7
SiO_2	%	**98.9**	**98.7**	**98.9**	**97.9**	**99.8**	**99.8**	**99.1**	**98.3**

Supplementary Table 1. **ICP-OES and EDS measurements of major elements in core PS1768-8.** Al_2O_3 concentrations of 0.5 and 0.6% are still in the range, which apparently do not affect the $\delta^{18}O$ signal[40]. The microscopical inspection of the samples with these Al_2O_3 concentrations does not indicate contamination by minerals.

Water depth (m)	A. antarctica % living	A. antarctica % juveniles	S. glacialis % living
PS2101-2			
0-50	17.22		0.5
50-100	22.54		
100-200	5.64		
200-400	17.63		
400-1000	1.11		
PS2103-3			
0-50	5.18	2.69	0.96
50-100	13.16	0.69	
100-300	10.21	3.4	
PS 2105-4			
0-50			16.79
50-100			29.74
100-200	1.19		9
200-400			2.43
400-1000			
PS2107-2			
0-50			1.06
50-100		1.91	
100-300	0.88	0.88	
300-400	2.19		
400-1000	1.33		

Supplementary Table 2. Vertical abundances of the radiolarian species *Actinomma antarctica* and *Spongotrochus glacialis* from four plankton stations. Data from the four stations in the Atlantic sector of the Southern Ocean are from ref. 25. Supplementary Figure 13 displays the location of the plankton stations. Both species were not encountered in the plankton hauls from the three northern station[25].

surface sediment stations

Surface Sediment Samples	Latitude	Longitude	rad size fraction	$\delta^{30}Si_{rad}$ [‰]	$\delta^{30}Si_{rad}$ [‰]	$\delta^{30}Si_{rad}$ [‰]	$\delta^{30}Si_{rad}$ [‰]	$\delta^{30}Si_{rad}$ AVG [‰]
**PS63/026-2**	50.5°S	01.5°E	>250 µm	1.21	1.37	1.41		**1.33**
**PS63/035-2**	55.5°S	00.5°E	>250 µm	0.74	0.85			**0.80**
**PS63/042-2**	58.5°S	00.5°E	>125 µm	0.74				**0.74**
**PS63/043-2**	59.5°S	00.5°E	>125 µm	0.86	0.99	0.71	0.6	**0.79**

water stations

Water Stations	Latitude	Longitude	$Si(OH)_4$ depth1 [µM l^{-1}]	$\delta^{30}Si$ depth1 [‰]	$\Delta\delta^{30}Si_{rad1}$ [‰]	$Si(OH)_4$ depth2 [µM l^{-1}]	$\delta^{30}Si$ depth2 [‰]	$\Delta\delta^{30}Si_{rad2}$ [‰]	$Si(OH)_4$ ODV annual (0-400 m) [µM l^{-1}]	$Si(OH)_4$ ODV annual (0-1000 m) [µM l^{-1}]	$Si(OH)_4$ ODV jan-mar (0-400 m) [µM l^{-1}]
**Large 6** (03/08/2008)	50.22°S	01.18°E	**31.2** (3-401m)	**2.01** (3-401m)	**-0.68** (3-401m)	**42.1** (3-999m)	**1.87** (3-999m)	**-0.54** (3-999m)	**30.1**	**45.1**	**29.1**
**Large 7** (03/14/2008)	55.14°S	00.03°E	**66.7** (4-300m)	**1.70** (4-300m)	**-0.91** (4-300m)	**74.7** (4-1002m)	**1.60** (4-1002m)	**-0.81** (4-1002m)	**67.6**	**79.8**	**69.9**
**Super 5** (03/16/2008)	57.32°S	00.02°E	**89.4** (29-400m)	**1.56** (29-400m)	**-0.82** (29-400m)	**97.96** (29-1003m)	**1.51** (29-1003m)	**-0.77** (29-1003m)	**81.5**	**94.4**	**81.5**
**Super 5** (03/16/2008)	57.32°S	00.02°E	**89.4** (29-400m)	**1.56** (29-400m)	**-0.77** (29-400m)	**97.96** (29-1003m)	**1.51** (29-1003m)	**-0.72** (29-1003m)	**83.9**	**97.3**	**83.6**

Supplementary Table 3. $\delta^{30}Si_{rad}$ values from surface sediment samples, $\delta^{30}Si_{Si(OH)4}$ and $Si(OH)_4$ values from nearby water stations and $\Delta\delta^{30}Si_{rad}$ estimations. $\Delta\delta^{30}Si_{rad}$ values are estimated from $\delta^{30}Si_{rad}$ values obtained from multicorer surface sediments (Fig. 1) and $\delta^{30}Si_{Si(OH)4}$ values measured at the nearby water stations[29] according to the equation: $\Delta\delta^{30}Si_{rad}=\delta^{30}Si_{rad}-\delta^{30}Si_{Si(OH)4}$ (ref. 21). Calculations were done for two water depth intervals, the upper ~300 to 400m and ~upper 1000m to cover the depth ranges of all radiolarian species included in the fractions. Obtained $\Delta\delta^{30}Si_{rad}$ values were compared to the $Si(OH)_4$ concentrations of both water depth intervals of each water station[29]. In addition, annual and summer (January-March) $Si(OH)_4$ concentrations at the surface sediment stations were averaged from two water depth intervals (0-400 m, 0-1000 m), which were taken from the Southern Ocean Atlas[33]. For the depth interval 0-1000 m only summer values were available. $\delta^{30}Si_{rad}$ AVG = averaged $\delta^{30}Si_{rad}$.

AVG $\delta^{30}Si_{diat}$ [‰]	$\delta^{30}Si_{rad}$ (>250µm) [‰]	$\delta^{30}Si_{rad}$ (125-250µm) [‰]	$\delta^{30}Si_{rad}$ (>125µm) [‰]	$\Delta\delta^{30}Si$ [‰]	$\delta^{30}Si(OH)_4$ [‰]	$Si(OH)_4$ [µM l^{-1}]	Fractions	Core
**Holocene**								
1.27				-1.1	2.37	0-25	diatom	PS1768-8
1.53				-1.1	2.64	0-20	diatom	PS1778-4
	1.03			-0.8	1.83	15-65	rad >250 µm	PS1768-8
	1.03			-1.2	2.23	0-25	rad >250 µm	PS1768-8
	1.03			-1.5	2.53	0-20	rad >250 µm	PS1768-8
		-0.24		-0.8	0.56	150-175	rad 125-250 µm	PS1768-8
		-0.24		-1.2	1.04	75-130	rad 125-250 µm	PS1768-8
		-0.24		-1.5	1.26	40-140	rad 125-250 µm	PS1768-8
			0.28	-0.8	1.08	65-130	rad >125 µm	PS1778-4
			0.28	-1.2	1.48	50-130	rad >125 µm	PS1778-4
			0.28	-1.5	1.78	15-65	rad >125 µm	PS1778-4
**Glacial**								
1.15				-1.1	2.25	0-25	diatom	PS1768-8
1.64				-1.1	2.74	0-20	diatom	PS1778-4
	0.54			-0.8	1.34	60-150	rad >250 µm	PS1768-8
	0.54			-1.2	1.74	40-80	rad >250 µm	PS1768-8
	0.54			-1.5	2.04	20-40	rad >250 µm	PS1768-8
		-0.67		-0.8	0.16	>170	rad 125-250 µm	PS1768-8
		-0.67		-1.2	0.56	~170	rad 125-250 µm	PS1768-8
		-0.67		-1.5	0.86	~110-170	rad 125-250 µm	PS1768-8
			-1.06	-0.8	-0.26	>170	rad >125 µm	PS1778-4
			-1.06	-1.2	0.14	>170	rad >125 µm	PS1778-4
			-1.06	-1.5	0.44	~170	rad >125 µm	PS1778-4

Supplementary Table 4. Parameters used for the estimation of Holocene and Glacial $\delta^{30}Si_{Si(OH)4}$ values compared to $Si(OH)_4$ values from field data. Indicated are $\delta^{30}Si$ values from diatom and radiolarian fractions of cores PS1768-8 and PS1778-5 averaged for the early Holocene (until 12 cal. ka BP) and last glacial period (~19-29 cal. ka BP). The calculation of $\delta^{30}Si_{Si(OH)4}$ from $\delta^{30}Si_{rad}$ data was performed with different fractionation offsets ($\Delta\delta^{30}Si_{rad}$= -0.8‰, $\Delta\delta^{30}Si_{rad}$= -1.2‰, $\Delta\delta^{30}Si_{rad}$= -1.5‰, see Methods). For the $\delta^{30}Si_{Si(OH)4}$ estimations from $\delta^{30}Si_{diat}$ data a diatom fractionation offset of -1.1‰ (ref. 24) was used. The obtained $\delta^{30}Si_{Si(OH)4}$ values were related to $\delta^{30}Si_{Si(OH)4}$ values from water stations and to their ranges in $Si(OH)_4$ concentrations[20,28,29] (Fig.4). Note that the $\delta^{30}Si_{Si(OH)4}$ values reflect a broad range in $Si(OH)_4$ concentrations as also indicated in Figure 4.

Depth (cm)	Age (yr)	Pointer definition
12.5	5172	$^{230}Th_{ex}$ constant flux modeling
54	9896	^{14}C, Calib7, Marine13 (Res 800 yr)
78	11093	^{14}C, Calib7, Marine13 (Res 800 yr)
142	13938	^{14}C, Calib7, Marine13 (Res 800 yr)
200	21000	upper 2 *E. antarctica* 1 peak
260	29000	mid increase *C. davisiana* b2 peak (MIS2/3 boundary)
310	37000	upper *C. davisiana* c1 low

Supplementary Table 5. Age pointers for PS1768-8. The AMS^{14}C dates from ref. 38 were converted to calendar years with CALIB 7.0 using the MARINE13 calibration[S12] with a reservoir age of 800 yr according to ref. S13. Pointers based on *Eucampia antarctica* and *Cycladophora davisiana* abundance fluctuations, both presenting established stratigraphic tools for the late Quaternary SO and calibrated with oxygen isotope stratigraphy[S5, S14-S18], represent an abundance maximum of *E. antarctica* (upper $2Ea_1$)[S16] at 200 cm, and the midpoint of the *C. davisiana* increase to its b peak[S15] at 260 cm, which marks the Marine Isotope Stage (MIS) 3/2 boundary[S18,S19] and the uppermost c1 low of *C. davisiana*[S15,S18]. For details on *E. antarctica* and *C. davisiana* abundance pattern see Supplementary Fig. 7. The pointer at 12.5 cm was defined by $^{230}Th_{ex}$ constant flux modeling considering an age model frame based on a combination of AMS ^{14}C dates and siliceous microfossil biofluctuation stratigraphies[50].

Depth (cm)	Age (yr)	Pointer definition
20	12000	upper end of *C. davisiana* b1 peak
40	14000	mid-way *C. davisiana* b-a
130	21000	mid *C. davisiana* b1 increase, upper *E. antarctica* peak
240	25000	mid *C. davisiana* b2 peak
350	29000	mid *C. davisiana* b2 incease (MIS2/3 boundary)

Supplementary Table 6. Age pointer for PS1778-5. Age assignments of pointers based on *E. antarctica* and *C. davisiana* abundance fluctuations[S14-S18] are from ref. 38. For details on *E. antarctica* and *C. davisiana* abundance pattern see Supplementary Fig. 8.

Station		Latitude	Longitude	δ^{30}Si [‰]	δ^{30}Si [‰]	δ^{30}Si [‰]	$\delta^{30}Si_{AVG}$ [‰]
PS2506-1	diatoms (10-40 µm)	47° 37.20 S	15° 25.40 W	1.61	1.64		**1.63**
	radiolarians (>125 µm)	51°24.50 S	25°42.00 E	0.84	0.9		**0.87**
PS63/30-3	diatoms (10-40 µm)	52° 59.55 S	0° 01.48 E	1.51	1.30	0.95	**1.25**

Supplementary Table 7. $\delta^{30}Si_{diat}$ and $\delta^{30}Si_{rad}$ values from surface sediments close to the core sites PS1768-8 and PS1778-5.

PS1768-8 Diatoms (10-40 µm) δ^{18}O (‰ vs. SMOW)					
Depth (cm)	Age [yrs]	δ^{18}O [‰]	δ^{18}O [‰]	δ^{18}O [‰]	**δ^{18}O$_{AVG}$ [‰]**
12.5	5172	43.64	43.18		**43.41**
23.5	6424	43.72	43.30		**43.51**
31.5	7335	43.72	43.23		**43.48**
38.5	8132	43.55	43.51		**43.53**
47.5	9156	43.76	43.65		**43.71**
58.5	10120	43.85	43.90		**43.88**
68.5	10619	43.67	43.90		**43.79**
91.5	11693	43.95	44.00		**43.98**
101.5	12138	43.29	44.12	44.95	**44.12**
112.5	12627	44.44	44.61		**44.53**
119.5	12938	44.06	44.18		**44.12**
129.5	13382	44.54	44.42		**44.48**
139.5	13827	44.57	44.23		**44.40**
149.5	14851	44.54	44.45		**44.50**
159.5	16069	44.67	44.13		**44.40**
169.5	17286	44.37	44.74		**44.56**
179.5	18504	44.73			**44.73**
191.5	19965	44.12	44.23		**44.18**
200.5	21067				
211.5	22533				
221.5	23867	44.19	44.69		**44.44**
231.5	25200	44.60	45.06		**44.83**
241.5	26533	43.83	43.82		**43.83**
251.5	27867	44.04	45.09		**44.57**
261.5	29240	44.57	44.50		**44.54**
271.5	30840	44.07	44.29		**44.18**

PS1768-8 Diatoms (10-40 µm) δ^{30}Si (‰ vs. NBS)					
Depth (cm)	Age [yrs]	δ^{30}Si [‰]	δ^{30}Si [‰]	δ^{30}Si [‰]	**δ^{30}Si$_{AVG}$ [‰]**
12.5	5172	1.19	1.33		**1.26**
23.5	6424	1.37	1.34		**1.36**
31.5	7335	1.27	1.19		**1.23**
38.5	8132	1.18	1.25		**1.22**
47.5	9156	1.43	1.39		**1.41**
58.5	10120	1.06	1.03		**1.05**
68.5	10619	1.15	1.25		**1.20**
91.5	11693	1.38	1.48		**1.43**
101.5	12138	1.15	1.18		**1.17**
112.5	12627	0.63	0.45		**0.54**
119.5	12938	0.83	0.99		**0.91**
129.5	13382	0.99	1.04		**1.02**
139.5	13827	0.77	1.01	0.40	**0.73**
149.5	14851	1.20	1.06		**1.13**
159.5	16069	1.06	1.53	1.23	**1.27**
169.5	17286	1.08	1.18	1.00	**1.09**
179.5	18504	0.78	0.70		**0.74**
191.5	19965	1.18	1.05		**1.12**
200.5	21067				
211.5	22533				
221.5	23867	1.17	1.03		**1.10**
231.5	25200	1.27	1.05		**1.16**
241.5	26533	1.31	1.10		**1.21**
251.5	27867	1.09	0.82		**0.96**
261.5	29240	0.70	0.68		**0.69**
271.5	30840	0.93			**0.93**

Supplementary Table. 8. Oxygen and silicon isotope data from diatom opal (10-40 µm fraction) in core PS1768-8.

PS1768-8 Radiolarians >250 µm		$\delta^{18}O$ (‰ vs. SMOW)			
Depth [cm]	Age [yrs]	$\delta^{18}O$ [‰]	$\delta^{18}O$ [‰]	$\delta^{18}O$ [‰]	**$\delta^{18}O_{AVG}$ [‰]**
12.5	5172	43.06	42.64		**42.85**
23.5	6424				
31.5	7335	43.49	43.84	43.74	**43.69**
38.5	8132	43.45	43.91		**43.68**
47.5	9156	43.34	44.42		**43.88**
58.5	10120	43.29	43.95		**43.62**
68.5	10619	43.80	43.71	43.79	**43.77**
91.5	11693	43.78	44.17		**43.98**
101.5	12138				
112.5	12627	44.06	44.26	44.13	**44.15**
119.5	12938	44.12	44.38		**44.25**
129.5	13382				
139.5	13827	44.01			**44.01**
149.5	14851	43.96	44.39		**44.18**
159.5	16069		44.42		**44.42**
169.5	17286	44.02	44.28		**44.15**
179.5	18504	43.25	44.34	43.93	**44.14**
191.5	19965	44.24	44.23		**44.24**
200.5	21067	43.59	43.89		**43.74**
211.5	22533	44.19	44.41		**44.30**
221.5	23867	43.87	43.90		**43.89**
231.5	25200	43.61	44.14	43.92	**43.89**
241.5	26533	44.59	44.58		**44.59**
251.5	27867	44.59	44.74		**44.67**
261.5	29240	44.93	45.30		**45.12**
271.5	30840	44.33	44.66		**44.50**

Supplementary Table 9. Oxygen isotope data from radiolarian opal of the >250 µm fraction from core PS1768-8.

**PS1768-8 Radiolarians >250 µm**		**δ^{30}Si (‰ vs. NBS)**			
Depth [cm]	Age [yrs]	δ^{30}Si [‰]	δ^{30}Si [‰]	δ^{30}Si [‰]	**$\delta^{30}Si_{AVG}$ [‰]**
12.5	5172	1.03	1.28		**1.16**
23.5	6424				
31.5	7335	1.30	0.99	1.10	**1.13**
38.5	8132	1.19	1.15		**1.17**
47.5	9156				
58.5	10120	0.90	0.75	0.88	**0.84**
68.5	10619	0.90	0.87		**0.89**
91.5	11693	1.04	1.00		**1.02**
101.5	12138				
112.5	12627	0.90	0.88		**0.89**
119.5	12938	0.85	0.92		**0.89**
129.5	13382				
139.5	13827	0.67			**0.67**
149.5	14851				
159.5	16069				
169.5	17286				
179.5	18504	0.46	0.50		**0.48**
191.5	19965	0.50	0.38		**0.44**
200.5	21067	0.64	0.51		**0.57**
211.5	22533	0.51	0.50	0.57	**0.53**
221.5	23867	0.22	0.45	0.71	**0.46**
231.5	25200	0.58	0.53	0.76	**0.62**
241.5	26533	0.53	0.44	0.57	**0.51**
251.5	27867	0.62	0.69		**0.66**
261.5	29240	0.47	0.36		**0.42**
271.5	30840	0.53	0.37		**0.45**

**PS1768-8 Radiolarians 125-250 µm**		**δ^{30}Si (‰ vs. NBS)**			
Depth [cm]	Age [yrs]	δ^{30}Si [‰]	δ^{30}Si [‰]	δ^{30}Si [‰]	**$\delta^{30}Si_{AVG}$ [‰]**
12.5	5172	-0.42	-0.47		**-0.45**
23.5	6424				
31.5	7335	-0.33	-0.46	-0.46	**-0.42**
38.5	8132	-0.38	-0.44		**-0.41**
47.5	9156				
58.5	10120	0.03	0.11		**0.07**
68.5	10619	-0.40	-0.27		**-0.34**
91.5	11693	0.07			**0.07**
101.5	12138				
112.5	12627				
119.5	12938				
129.5	13382				
139.5	13827				
149.5	14851				
159.5	16069				
169.5	17286				
179.5	18504	-0.40	-0.80		**-0.60**
191.5	19965	-0.46	-0.48		**-0.47**
200.5	21067	-0.42	-0.32	-0.54	**-0.43**
211.5	22533	-0.54	-0.70		**-0.62**
221.5	23867	-0.62			**-0.62**
231.5	25200	-0.76	-0.73		**-0.75**
241.5	26533	-0.67	-0.74		**-0.71**
251.5	27867	-0.90			**-0.90**
261.5	29240	-0.97	-0.80		**-0.89**
271.5	30840	-0.56	-0.91		**-0.74**

Supplementary Table 10. Silicon isotope data from radiolarian opal of the >250 µm and 125-250µm fractions from core PS1768-8.

PS1778-5 Diatoms (10-40 µm) $\delta^{18}O$ (‰ vs. SMOW)

Depth (cm)	Age[yrs]	$\delta^{18}O$ [‰]	$\delta^{18}O$ [‰]	$\delta^{18}O$ [‰]	$\delta^{18}O$ [‰]	$\delta^{18}O$ [‰]	**$\delta^{18}O_{AVG}$ [‰]**
0.5	10050	41.95	41.66	41.52			**41.71**
7.0	10700	41.99	42.23	41.85			**42.02**
17.0	11700	42.78	42.58				**42.68**
27.0	12700	42.99	42.71				**42.85**
37.0	13700	43.47	43.84				**43.66**
46.5	14506	43.51	43.53				**43.52**
56.5	15283	43.42	43.03				**43.23**
66.5	16061	44.25	44.13				**44.19**
77.0	16878	44.28	43.99				**44.14**
87.0	17656	44.36	44.31				**44.34**
97.0	18433	43.85	43.60	43.74			**43.73**
107.0	19211	43.83	44.06				**43.95**
117.0	19989	43.49	43.39				**43.44**
127.0	20767	44.09	43.98				**44.04**
137.0	21255	44.20	44.17				**44.19**
147.0	21618	44.03	43.93				**43.98**
157.0	21982	44.26	43.88	43.54			**43.89**
167.0	22345	43.69	44.13	43.47			**43.76**
180.5	22836	44.14	44.14				**44.14**
187.5	23091	44.01	44.21	44.11			**44.11**
197.0	23436	43.75	43.81	43.87			**43.81**
207.5	23818	44.00	44.20				**44.10**
218.0	24200	43.44	44.07	43.42	44.28	43.08	**43.66**
228.0	24564	43.91	43.60	43.35			**43.62**
238.0	24927	43.78	43.60	44.19	43.85		**43.86**
247.5	25273	44.20	44.13				**44.17**
257.5	25636	44.40	44.68				**44.54**
267.5	26000	44.40	*43.32*	44.46			**44.43**
280.5	26473	44.56	44.02	44.40			**44.33**
287.5	26727	43.47	43.67				**43.57**
297.5	27091	44.47	44.69				**44.58**
307.0	27436	43.58	43.95	43.75			**43.88**
318.0	27836	43.64	44.29	44.02			**43.98**
327.5	28182	43.54	43.37				**43.46**
337.0	28527	44.00	44.39	43.62			**44.00**
347.0	28891	44.30	43.76	44.03			**44.03**
357.0	29255	44.26	44.18				**44.22**
367.0	29618	44.05	44.05				**44.05**
381.0	30127	43.89	43.67	43.81			**43.79**

PS1778-5 Diatoms (10-40 µm) $\delta^{30}Si$ (‰ vs. NBS)

Depth (cm)	Age	$\delta^{30}Si$ [‰]	$\delta^{30}Si$ [‰]	$\delta^{30}Si$ [‰]	$\delta^{30}Si$ [‰]	$\delta^{30}Si$ [‰]	**$\delta^{30}Si_{AVG}$ [‰]**
0.5	10050	1.41	1.47				**1.44**
7.0	10700	1.56	1.52	1.45	1.65		**1.55**
17.0	11700	1.57	1.57	1.68	1.59		**1.60**
27.0	12700	1.57	1.56				**1.57**
37.0	13700	1.56	1.71	1.47	1.66		**1.60**
46.5	14506	1.63	1.69	1.60			**1.64**
56.5	15283	1.59	1.72	1.62	1.57		**1.63**
66.5	16061	1.61	1.62	1.56			**1.60**
77.0	16878	1.63	1.69	1.59			**1.64**
87.0	17656	1.68	1.78	1.52			**1.66**
97.0	18433	1.59	1.58				**1.59**
107.0	19211	1.56	1.60				**1.58**
117.0	19989	1.60	1.59				**1.60**
127.0	20767	1.74	1.71	1.64			**1.70**
137.0	21255	1.63	1.63				**1.63**
147.0	21618	1.68	1.64	1.65			**1.66**
157.0	21982	1.68	1.53	1.70	1.66		**1.64**
167.0	22345	1.69	1.65	1.60	1.77		**1.68**
180.5	22836	1.61	1.80	1.57	1.76		**1.69**
187.5	23091	1.60	1.78	1.65			**1.68**
197.0	23436	1.78	1.68	1.65			**1.70**
207.5	23818	1.65	1.65				**1.65**
218.0	24200	1.63	1.69	1.64	1.52	1.70	**1.64**
228.0	24564	1.58	1.60	1.51	1.72		**1.60**
238.0	24927	1.57	1.56	1.63	1.65		**1.60**
247.5	25273	1.62	1.60	1.64			**1.62**
257.5	25636	1.72	1.58	1.62			**1.64**
267.5	26000	1.54	1.57				**1.56**
280.5	26473	1.75	1.61	1.69			**1.68**
287.5	26727	1.58	1.50	1.53			**1.54**
297.5	27091	1.62	1.61	1.50			**1.58**
307.0	27436	1.68	1.66	1.64	1.64		**1.66**
318.0	27836	1.75	1.70	1.93	1.86		**1.81**
327.5	28182	1.68	1.62	1.58			**1.63**
337.0	28527	1.75	1.54	1.80	1.61		**1.68**
347.0	28891	1.52	1.57				**1.55**
357.0	29255	1.62	1.60	1.50			**1.57**
367.0	29618	1.71	1.64	1.62			**1.66**
381.0	30127	1.58	1.56				**1.57**

Supplementary Table 11. Oxygen and silicon isotope data from diatom opal (10-40 µm fraction) in core PS1778-5.

PS1778-5 Radiolarians (>125 µm) $\delta^{18}O$ (‰ vs. SMOW)

Depth (cm)	Age[yrs]	$\delta^{18}O$ [‰]	$\delta^{18}O$ [‰]	$\delta^{18}O$ [‰]	$\delta^{18}O$ [‰]	$\delta^{18}O_{AVG}$ [‰]
0.50	10050	42.42	42.41	42.46		**42.43**
7.00	10700	43.21	42.94	42.73		**42.96**
17.00	11700	44.00	43.75	42.87	43.23	**43.46**
27.00	12700	44.06	43.71	43.69		**43.82**
37.00	13700	43.50	43.53			**43.52**
46.50	14506	44.64	43.87	43.56		**44.02**
56.50	15283	44.16	43.98	43.50		**43.88**
66.50	16061	43.22	42.77			**43.00**
77.00	16878	44.04				**44.04**
87.00	17656	43.46	44.60			**44.03**
97.00	18433	43.60	43.95			**43.78**
107.00	19211	43.86	44.38	44.10		**44.11**
117.00	19989	44.13	43.79	44.19		**44.04**
127.00	20767	43.96	44.23	43.71		**43.97**
137.00	21255	43.33	44.27			**43.80**
147.00	21618	44.02	43.27			**43.65**
157.00	21982	44.36	44.72	43.70		**44.26**
167.00	22345	44.87	44.67			**44.77**
180.50	22836	44.02	44.70			**44.36**
187.50	23091	44.43	44.94			**44.69**
197.00	23436	45.29	44.99			**45.14**
207.50	23818	44.36				**44.36**
218.00	24200	43.70	43.35			**43.53**
228.00	24564	44.85	44.76	44.68	44.42	**44.68**
238.00	24927	44.12	44.23	44.10		**44.15**
247.50	25273	45.05	44.02	44.70		**44.59**
257.50	25636	44.56	44.26	44.00		**44.27**
267.50	26000	44.91	44.44	44.22		**44.52**
280.50	26473	44.00	44.82			**44.41**
287.50	26727	43.97	44.12			**44.05**
297.50	27091	43.80				**43.80**
307.00	27436	44.85	44.34	44.24		**44.48**
318.00	27836	44.73	44.51	44.15		**44.46**
327.50	28182	44.52	44.09	43.70		**44.10**
337.00	28527	44.74	44.57			**44.66**
347.00	28891	44.51	44.36			**44.44**
357.00	29255	44.28	43.82			**44.05**
367.00	29618	44.09	43.80			**43.95**
381.00	30127	43.69	44.12			**43.91**

PS1778-5 Radiolarians (>125 µm) $\delta^{30}Si$ (‰ vs. NBS)

Depth (cm)	Age[yrs]	$\delta^{30}Si$ [‰]	$\delta^{30}Si$ [‰]	$\delta^{30}Si$ [‰]	$\delta^{30}Si$ [‰]	$\delta^{30}Si_{AVG}$ [‰]
0.50	10050	0.09	0.28	0.16		**0.18**
7.00	10700	0.74	0.64			**0.69**
17.00	11700	0.24	-0.16	0.13	-0.36	**-0.04**
27.00	12700	-0.09	0.82	-0.05	0.50	**0.30**
37.00	13700	-0.63	-0.56			**-0.60**
46.50	14506	-1.40	-1.66	-1.47		**-1.51**
56.50	15283	-1.60	-1.53			**-1.57**
66.50	16061	-0.37	-0.27			**-0.32**
77.00	16878	-0.34				**-0.34**
87.00	17656	-1.73				**-1.73**
97.00	18433	-0.93				**-0.93**
107.00	19211	-1.64	-2.09			**-1.87**
117.00	19989	-0.47	-1.13	-1.76		**-1.12**
127.00	20767	-1.40	-1.41			**-1.41**
137.00	21255	-0.87	-1.15			**-1.01**
147.00	21618	0.21				**0.21**
157.00	21982	-0.66	-0.94			**-0.80**
167.00	22345	-0.77	-0.71			**-0.74**
180.50	22836	-1.09	-1.17			**-1.13**
187.50	23091	-0.67				**-0.67**
197.00	23436	-1.24				**-1.24**
207.50	23818	-1.12				**-1.12**
218.00	24200	-0.53	-0.58	-1.42	-1.06	**-0.90**
228.00	24564	-0.83	-1.11			**-0.97**
238.00	24927	-1.47	-1.01			**-1.24**
247.50	25273	-1.61				**-1.61**
257.50	25636	-0.82	-1.53			**-1.18**
267.50	26000	-0.51				**-0.51**
280.50	26473	-1.14	-1.28			**-1.21**
287.50	26727	-0.60	-1.41			**-1.01**
297.50	27091	-1.08				**-1.08**
307.00	27436	-1.48	-1.32			**-1.40**
318.00	27836	-0.75	-1.02			**-0.89**
327.50	28182	-1.43	-0.63			**-1.03**
337.00	28527	-1.80	-1.44			**-1.62**
347.00	28891	-0.97	-1.13			**-1.05**
357.00	29255	-0.93	-1.07			**-1.00**
367.00	29618	-1.00	-1.43	-0.64		**-1.02**
381.00	30127	-0.76	-0.86			**-0.81**

Supplementary Table 12. Oxygen and silicon isotope data from radiolarian opal of the >125 µm fraction from core PS1778-5.

Supplementary References

1. Esper, O. & Gersonde, R. Quaternary surface water temperature estimations: New diatom transfer functions for the Southern Ocean. *Palaeogeography Palaeoclimatology Palaeoecology* **414**, 1-19. doi:10.1016/j.palaeo.2014.08.008 (2014).

2. Diekmann *et al.* in *The South Atlantic in the late Quaternary* (eds Wefer, G., Mulitza, S., Rathmeyer, V.) 375-399 (Springer, 2004).

3. Niebler, H. S. Stable isotope record of foraminifera from South Atlantic sediments with reconstruction of paleotemperatures and paleosalinities. doi:10.1594/*PANGAEA*.835327 (1995).

4. WAIS Divide Project Members. Onset of deglacial warming in West Antarctica driven by local orbital forcing. *Nature* **500**, 440-444 (2013).

5. Charles, C. D. *et al.* Biogenic opal in Southern Ocean sediments over the last 450.000 years: implications for surface water chemistry and circulation. *Paleoceanography* **6**, 697-728 (1991).

6. Bianchi, C. & Gersonde, R. Climate evolution at the last deglacial: The role of the Southern Ocean. *Earth and Planetary Science Letters* **228**, 407-424 (2004).

7. Charles, C. D. *Late Quaternary ocean chemistry and climate change from an Antarctic deep sea sediment perspective. PhD thesis* (Columbia University, N.Y., 1991).

8. Labeyrie, L. D., Pichon, J. J. Labracherie, M., Ippolito, P. Duprat, J. & Duplessy, J. C. Melting history of Antarctica during the past 60,000 years. *Nature* **322**, 701-706 (1986).

9. Brathauer, U. & Abelmann, A. Late Quaternary variations in sea surface temperatures and their relationship to orbital forcing recorded in the Southern ocean (Atlantic sector). *Paleoceanography* **14**, 135-148 (1999).

10. Hurrell, J. W., Hack, J. J., Shea, D., Caron, J. M. & Rosinski, J. A new sea surface temperature and sea ice boundary dataset for the Community Atmosphere Model. *J. Climate* **21**, 5145–5153 (2008). doi: http://dx.doi.org/10.1175/2008JCLI2292.1

11. Schlitzer, R. Ocean Data View. http://odv.awi.de (2013).

12. Stuiver, M., Reimer, P. J., & Reimer, R. W. CALIB 5.0. (*WWW program and documentation*) (2005).

13. Bard, E. Correction of accelerator mass spectrometry ^{14}C ages measured in planktonic foraminifera: paleoceanographic implications. *Paleoceanography* **3**, 635-645 (1988).

14. Morley, J. J., Hays, J. D. & Robertson, J. H. Stratigraphic framework of the late Pleistocene in the northwest Pacific Ocean. *Deep Sea Res.* **29**, 1485-1499 (1982).

15. Hays, J. D., Imbrie, J. & Shackleton, N. J. Variations in the Earth's Orbit: Pacemaker of the Ice Ages. *Science* **194**, 1121-1132 (1976)

16. Burckle, L. H. & Cooke, D. W. Late Pleistocene *Eucampia antarctica* abundance stratigraphy in the Atlantic sector of the Southern Ocean. *Micropaleontology* **29**, 6-10 (1983).

17. Burckle. L. H. & Burak, R. Relative abundance of *Eucampia antarctica* as a close proxy to $\delta^{18}O$ in upper Quaternary sediments of the Southern Ocean. *Pact* **50**, I.1, 15-22 (1996).

18. Brathauer, U., Abelmann, A., Gersonde, R., Niebler, S. & Fütterer, D. K. Calibration of *Cycladophora davisiana* events versus oxygen isotope stratigraphy in the subantarctic Atlantic Ocean – A stratigraphic tool for carbonate-poor Quaternary sediments. *Marine Geology* **175,** 167-181 (2001).

19. Martinson, D. G., Pisias, N. G., Hays, J. D., Imbrie, J. D., Moore, T. C. & Shackleton, N. J. Age dating and the orbital theory of the ice ages: development of a high-resolution 0 to 300,000-year chronostratigraphy. *Quaternary Research*, **27**, 1-29 **(**1987).
